# Two‐Dimensional Fluorinated Graphene: Synthesis, Structures, Properties and Applications

**DOI:** 10.1002/advs.201500413

**Published:** 2016-03-02

**Authors:** Wei Feng, Peng Long, Yiyu Feng, Yu Li

**Affiliations:** ^1^ School of Materials Science and EngineeringTianjin UniversityTianjin300072P.R China; ^2^ Collaborative Innovation Center of Chemical Science and Engineering (Tianjin)Tianjin300072P.R China; ^3^ Key Laboratory of Advanced Ceramics and Machining TechnologyMinistry of EducationTianjin300072P.R China; ^4^ Tianjin Key Laboratory of Composite and Functional MaterialsTianjin300072P.R China

**Keywords:** C–F bonds, F/C ratio, fluorinated graphene, fluorographene

## Abstract

Fluorinated graphene, an up‐rising member of the graphene family, combines a two‐dimensional layer‐structure, a wide bandgap, and high stability and attracts significant attention because of its unique nanostructure and carbon–fluorine bonds. Here, we give an extensive review of recent progress on synthetic methods and C–F bonding; additionally, we present the optical, electrical and electronic properties of fluorinated graphene and its electrochemical/biological applications. Fluorinated graphene exhibits various types of C–F bonds (covalent, semi‐ionic, and ionic bonds), tunable F/C ratios, and different configurations controlled by synthetic methods including direct fluorination and exfoliation methods. The relationship between the types/amounts of C–F bonds and specific properties, such as opened bandgap, high thermal and chemical stability, dispersibility, semiconducting/insulating nature, magnetic, self‐lubricating and mechanical properties and thermal conductivity, is discussed comprehensively. By optimizing the C–F bonding character and F/C ratios, fluorinated graphene can be utilized for energy conversion and storage devices, bioapplications, electrochemical sensors and amphiphobicity. Based on current progress, we propose potential problems of fluorinated graphene as well as the future challenge on the synthetic methods and C‐F bonding character. This review will provide guidance for controlling C–F bonds, developing fluorine‐related effects and promoting the application of fluorinated graphene.

## 1 Introduction

Since Andre Geim and Kostya Novoselov first isolated high‐quality few‐atom‐thick nanosheets (including single‐layer) from graphite, the ability to prepare graphene and its derivatives have triggered intense research in two‐dimensional nanomaterials all over the world.^1^ Subsequently, graphene‐based materials receive much attention in nanotechnology because of their extraordinary properties, such as an ultrahigh theoretical specific surface area (2630 m^2^ g^−1^), exceptional charge carrier mobility (200 000 cm^2^ V^−1^ s^−1^), high thermal conductivity (≈5000 W m^−1^ K^−1^), high optical transmittance (≈97.7%).^2^ Despite these aforementioned superiorities, pristine graphene suffers from several shortcomings including structural defects, chemical inertness and a zero bandgap. Thus, many functionalization methods such as chemical bonding, loading or generating functional groups or free radicals on graphene (or its derivatives) have been utilized to improve structural integrity, surface activity and processability.^3^ The functionalization not only inherits unique carbon conjugated structures but also brings about a promise to alter the graphene's properties including dispersion, orientation, interaction and electronic properties.^4^

Graphene oxide (GO)^5^ and halogenated graphene (CX*_m_*, X = F,Cl, Br, or I),[[qv: 4a]],^6^ typical members of graphene derivatives including fluorographane^7^ and thiofluorographene,^8^ have thousands of oxygen functional groups or halogen atoms on carbon nanosheets with the transition of carbon atoms from sp^2^ to sp^3^ hybridization. The chemical modification endows graphene with many excellent properties, such as good dispersion in organic/water solvents, chemical activity on the surface via functional groups and tunable electronic properties, such as bandgap opening, charge transfer density and work functions. Despite numerous studies, GO and halogenated graphene continue to have several problems as follows: (1) GO has a variety of chemical bonds containing carboxyl, carbonyl, hydroxyl, lactone, and epoxide at graphene edge and basal‐plan. The amount and concentration of these functional groups are not controlled. (2) Halogenated graphene usually is a mixture of nanosheets with different degrees of substitution and different halogen‐carbon bonds. (3) GO and halogenated graphene show less chemical and thermal stability than graphene as a result of a great amount of defects or substituents on the surface, especially for brominated‐ and iodine‐doped graphene. The representative characteristics of halogenated graphene were shown in **Table** **1**.^9^

**Table 1:** The empirical characteristics of halogenated graphene^9^

	CF*_x_*	CCl*_x_*	CBr*_x_*	CI*_x_*
Value of *x*	≈0–1.12	≈0–0.43	≈0–0.050	≈0–0.031
Synthesis methods	Fluorination of graphene Exfoliation methods	Chlorination of graphene	Modification by bromine	Doping by iodine
Calculated bandgap when *x* = 1	≈3.1 eV	≈0.9 eV	Almost no bandgap	—
Stability at room temperature	Stable	unstable	unstable	unstable
C–X in IR	≈1050–1250 cm^−1^	≈790 cm^−1^	≈600 cm^−1^	≈745 cm^−1^
C–X in XPS	≈684–689 eV (F 1s)	≈200.5 eV (Cl 2p_3/2_) ≈202.0 eV (Cl 2p_1/2_)	≈71 eV (Br 3d) ≈184.0 eV (Br 3p_3/2_) ≈191.0 eV (Br 3p_1/2_)	≈619.5 eV (I 3d_3/2_) ≈631.9 eV (I 3d_5/2_)

Fluorinated graphene is regarded as the two‐dimensional basic structural element of fluorinated graphite first synthesized by Ruff et al. in 1934.^10^ Subsequently, fluorinated graphite recevies much attention in self‐cleaning, solid lubricants, superhydrophobic coating, and the electrode of electrochemical cell because of its extremely low surface energy, good chemical and thermal stabilities, and high electromotive force (4.57 V at 25 °C calculated by thermodynamic data) in lithium‐fluorinted graphite battery.^11^ A typical method of preparing fluorinated graphite is the fluorination in fluorine‐containing atmosphere, and the F/C ratios and the C‐F bonds (covalent, semi‐ionic or ionic) usually depend on the fluorination conditions including the pressure, temperature and treatment time in fluorine‐containing atmosphere. Moreover, 2D fluorinated graphene with single or few layers can be obtained by exfoliating fluorinated graphite via mechanical or liquid phase exfoliation methods. High quality fluorinated graphene offers a great potential for modulating various properties by controlling the microstructures (layer, size and surface chemistry).

Fluorinated graphene (CF*_x_*, *x* ≈ 0–1.12), which is a stable and wide‐bandgap nanosheet in which a certain amount of C atoms is covalently bonded to F atoms, becomes a rising star of graphene derivatives because of its outstanding properties, such as a large negative magnetic resistance (a factor of 40 in a 9 T field), a wide optical bandgap (3.8 eV) and a high room‐temperature resistance (>10 GΩ).[[qv: 4a]],^12^ Fluorographene (fully fluorinated graphene, CF) is defined by Rahul R. Nair[[qv: 4a]] as a carbon monofluoride of graphene with the F/C ratio of 1.0, which is also introduced or accepted by many groups.^13^

Compared with other derivatives, fluorinated graphene shows many unique properties because of the formation of various types of C‐F bonds. First, because the F atoms has a higher electronegativity (4.0) than C (2.5), H (2.2), and O atoms (3.4), fluorinated graphene show great potential for using as an atomically thin insulator or a tunnel barrier based on the heterostructure.[[qv: 4a]] Second, because of the difference in electronegativity (1.5) between C and F atoms, fluorinated graphene exhibits several C‐F bonding characters from ionic, semi‐ionic to covalent bonds controlled by the fluorination conditions.^14^ The C‐F bonding character depends on the fluorination levels according to theoretical calculation.^15^ Third, fluorinated graphene is regarded as an excellent cathode material for high‐energy lithium batteries because of its ability to electrochemically store and release high‐density energy (theoretical energy density of Li/CF_1.0_ is 2162 Wh kg^−1^).^16^ Thus, a Li/CF*_x_* battery shows high energy densities, good chemical stability, a long‐term shelf life (>10 years) and minimal (<10%) self‐discharge.^17^ In particular, the theoretical specific capacity of Li/CF*_x_* (*x* = 1) is 865 mAh g^−1^ with an average discharge potential between 4.5 and 5 V for a purely ionic C–F bond.^18^ Fourth, covalent C‐F bonds show a high response to biological signals because of the high orientation and polarity of the C‐F bond.^19^ Thus, fluorinated graphene can be developed for various biological applications, such as promoting neuro‐induction of stem cells[[qv: 19b]] or as a single multimodal material for magnetic resonance imaging.^20^ Finally, C‐F bonds on the nanosheets greatly increase the hydrophobicity with an extreme low surface energy resulting in a super‐hydrohophic or amphiphilic film.^21^

**Figure 1:**
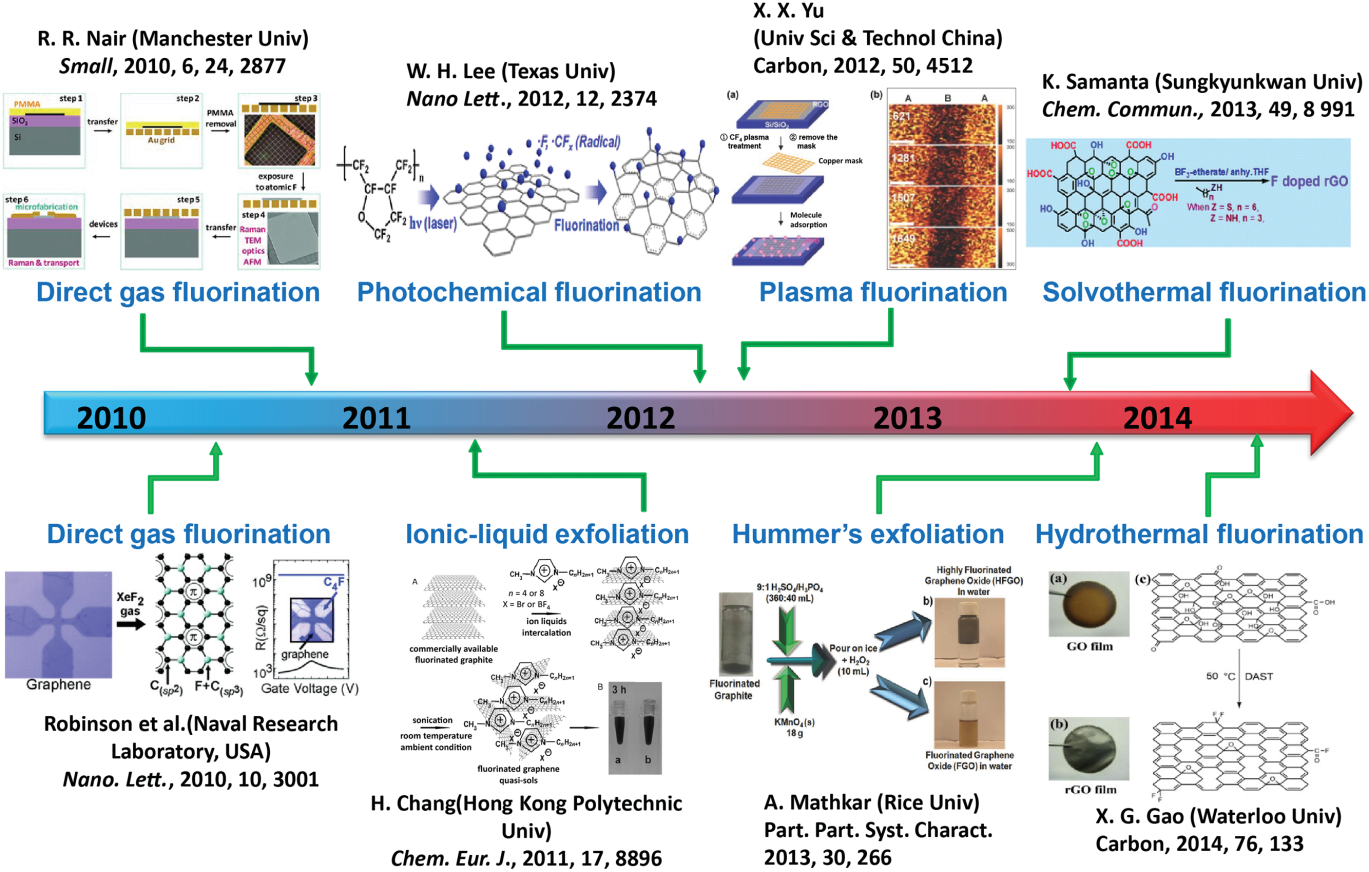
Timeline showing recent synthetic methods regarding fluorinated graphene. Reproduced with permission.[[qv: 4a]] Reproduced with permission.[[qv: 12a]] Copyright 2010, American Chemnical Society. Reproduced with permission.[[qv: 24a]] Copyright 2014, Elsevier. Reproduced with permission.[[qv: 24b]] Copyright 2013, Royal Society of Chemistry. Reproduced with permission.[[qv: 25a]] Copyright 2012, American Chemical Society. Reproduced with permission.[[qv: 26a]]

Recently, much progress has been made on the preparation and control of C‐F bonding characters, F/C ratios (the F/C ratio is defined as the mole ratio of fluorine to carbon) and configurations of fluorinated graphene. The brief roadmap of a synthetic strategy of fluorinated graphene is shown in **Figure** **1**. However, a comprehensive review about the relationship between C‐F bonding character and various properties of fluorinated graphene has not yet been reported. In this review, we present the recent progress and advances on synthesis methods, C‐F bonding character, properties (bandgap, optical properties, stability, electronic conductivity, dispersibility, magnetic, tribological, mechanical (micromechanical) properties and thermal conductivity) and applications in energy conversion and storage devices, biological devices, quantum dots, supercapacitors and amphiphilic coating of fluorinated graphene. This review provides guidance for regulating a variety of properties and performances of fluorinated graphene based on designing and controlling its C‐F bonding character, F/C ratios and configuration. A strategy of structural design, potential problems and present/future challenges of fluorinated graphene are also proposed.

## 2 Methods for Synthesizing Fluorinated Graphene

The methods for synthesizing fluorinated graphene or fluorographene are mainly classified into two groups: fluorination and exfoliation methods. Fluorination mainly include direct gas‐fluorination,[[qv: 4a]],[[qv: 12a]],[[qv: 13b]],[[qv: 21a]],^22^ plasma fluorination,^23^ hydrothermal fluorination,^24^ and photochemical/electrochemical synthesis.^25^ Exfoliation methods includes sonochemical exfoliation,[[qv: 9a]],^26^ modified Hummer's exfoliation,^20^,[[qv: 21b]] and thermal exfoliation.^27^ F/C ratios of fluorinated graphene prepared by different methods are summarized in **Table** **2**.

**Table 2:** Synthetic methods and preparation conditions for fluorinated graphene

	Synthetic method		F/C ratios	Reaction temperature	Reaction time	Ref
	Graphene‐based materials	Fluorine agents/exfoliation solvents				
Direct gas‐fluorination	Graphene membranes	XeF_2_	≈0–1.00	70 °C	≈1h–2 weeks	[[qv: 4a]]
	Graphene films	XeF_2_	≈0–0.25 for single‐sided fluorination	30 °C	≈30–1200 s	[[qv: 12a]]
			≈0–1.00 for double‐sided fluorination			
	Graphene sheets	XeF_2_	≈0–1.00	350 °C	≈1–5 day	[[qv: 13a]]
	GO	F_2_	≈0–1.02	From RT to 180 °C	20 min	[[qv: 22a]]
	Highly ordered pyrolytic graphite	F_2_	≈0.70	600 °C	36–48 h	[[qv: 22c]]
Plasma fluorination	CVD graphene	SF_6_ plasma	—	RT	6 s	[[qv: 23a]]
	Epitaxial graphene	SF_6_ plasma	≈0.10	RT	30 s	[[qv: 23g]]
	GO	CF_4_ plasma	0.17–0.27	RT	5–20 s	[[qv: 23k]]
	Graphene sheets	CF_4_ plasma	—	RT	3–20 min	[[qv: 23i]]
	CVD graphene	CF_4_ plasma	0.04–0.18	RT to 200 °C	1–20 min	[[qv: 23e]]
	Graphene	Ar/F_2_ plasma	0.17 for 3 min	RT	0.5–30 min	[[qv: 23h]]
Hydrothermal fluorination	GO dispersion	HF (40 wt%)	0.11–0.48	150–180 °C	10–30 h	[[qv: 24c]]
	GO films	diethylaminosulfur trifluoride	0.04–0.05	0 °C or RT or 50 °C	17 h	[[qv: 24a]]
	GO	anhydrous BF_3_‐etherate	0.39	60 °C	24 h	[[qv: 24b]]
Photochemical synthesis	CVD graphene	CYTOP	—	RT	—	[[qv: 25a]]
	GO	HF aqueous solution	0.33	RT	48 h	[[qv: 25c]]
Electrochemical synthesis	Graphite	hydrofluoric acid aqueous solution (50 wt%)	≈0.10	RT	6000 s	[[qv: 25b]]
Sonochemical exfoliation	Fluorinated graphite	sulffolane	1.00	50 °C	1 h	[[qv: 9a]]
	Fluorinated graphite	1‐butyl‐3‐methylimidazolium bromide	0.25 or 0.50	RT	3 h	[[qv: 26a]]
	Fluorinated graphite	NMP	0.78–0.31	RT	16–100 h	[[qv: 26b]]
	Fluorinated graphite	Chloroform or acetonitrile	≈0.90	RT	6 h	[[qv: 26c]]
	Fluorinated graphite	cetyl‐trimethyl‐ammonium bromide and dopamine	0.25	RT	10 min	[[qv: 26d]]
Modified Hummer's exfoliation	Graphite fluorinated polymer	H_2_SO_4_/H_3_PO_4_	0.07–0.36	50 °C	2 h	[[qv: 21b]]
Thermal exfoliation	Fluorinated graphite (CF_0.57_)	—	0.03–0.40	480 °C	30 min	27

### 2.1 Fluorination Methods

#### 2.1.1 Direct Gas‐Fluorination

**Figure 2:**
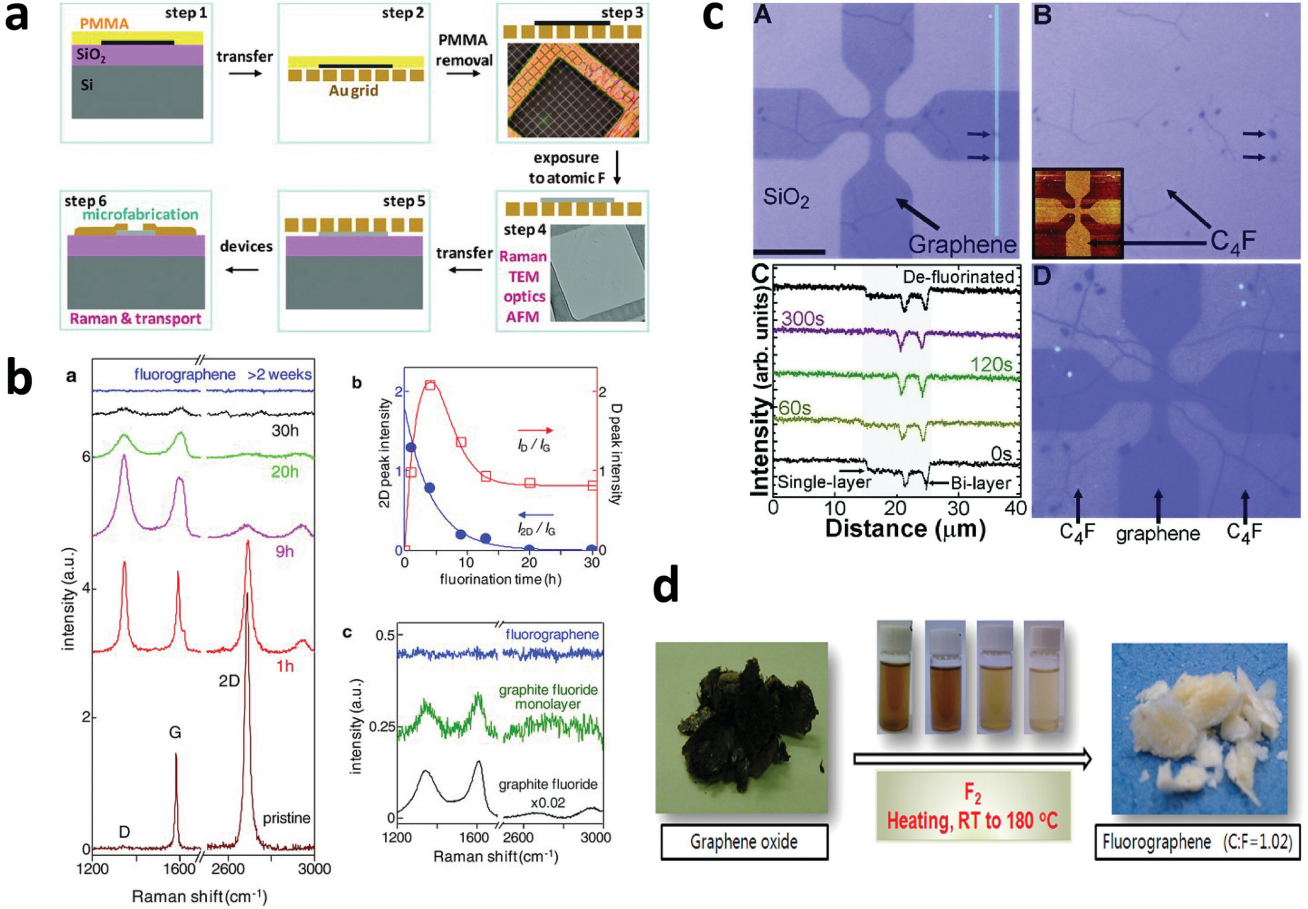
a) Various steps involved in the fluorination of grapheme (PMMA‐poly(methyl methacrylate)). b) Raman signatures of fluorinated grapheme (PMMA‐poly(methyl methacrylate)). Reproduced with permission.[[qv: 4a]] c) Optical changes of graphene upon single‐side fluorination. Reproduced with permission.[[qv: 12a]] Copyright 2010, American Chemical Society. d) Scheme for preparing fluorographene by direct‐heating fluorination of graphene‐oxide. Reproduced with permission.[[qv: 22a]] Copyright 2013, American Chemical Society.

Fluorographene was prepared by Nair et al.[[qv: 4a]] using XeF_2_ gas to treat a graphene film at 70 °C (**Figure** **2**a). The fluorination using XeF_2_ gas is one of widely used technique to prepare fluorographene with different fluorinated structures becuase of mild and controllable process. The resultant fluorographene showed high thermal stability up to 400 °C even in an atmospheric environment. Subsequently, the fluorination process was investigated by Raman spectroscopy (Figure 2b). An increase in the D band at 1350 cm^−1^ and a decreased 2D band at 2680 cm^−1^ indicated that the fluorination degree of graphene increased with a long XeF_2_ treatment time. Fluorographene was obtained until all D, 2D, and G bands disappeared.[[qv: 4a]] Furthermore, fluorographene was also prepared by fluorinating graphene grown by chemical vapor deposition (CVD) on the Si substrate using XeF_2_ gas at room temperature.[[qv: 12a]] Fluorographene showed a dominant stoichiometry of C_1.0_F_1.0_ and a high F/C ratio of graphene film on both the front and back surface because of the effective etching on the Si substrate by XeF_2_ gas. This effect was confirmed by fluorinated graphene (CF_0.25_) on Cu foils because the Cu substrate cannot be etched by XeF_2_ (Figure 2c). Raman spectra showed the D, D' and D+D' peak, while G peak was broadened by the exposure to XeF_2_. This result indicated the introduction of a high degree of structural disorder in the fluorinated graphene.[[qv: 12a]] In addition, fluorographene was also synthesized by treating graphene sheets in XeF_2_ at 350 °C for 1 and 5 days in an inert atmosphere.[[qv: 13a]] Despite a tunable F/C ratio, the large‐scale production of fluorographene is restricted by the high‐temperature fluorination and the expensive XeF_2_.

Fluorine gas (F_2_) is another important fluorination agent to prepare fluorinated graphene because of its high reactivity. Fluorinated graphene with different F/C ratios was synthesized by Wang et al.[[qv: 22a]] GO was treated by F_2_ at a low temperature (from room temperature to 180 °C) (Figure 2d). The F/C ratios (0.65, 0.84, and 1.02) of fluorinated graphene could be controlled by the concentration (2%, 5%, and 10%) of F_2_ in a mixture of F_2_ and N_2_ gas. In addition, Cheng et al.[[qv: 22c]] prepared fluorinated graphene by the exfoliation of fluorinated highly oriented pyrolitic graphite (HOPG), which was treated using 1 atm F_2_ at a high temperature of 600 °C. The resultant few‐layer fluorinated graphene showed a high F/C ratio (CF_0.7_). Interestingly, Sofer et al.[[qv: 22e]] also presented an easy and weighable method for the fluorination of Hummers GO and Staudenmaier GO in 20% F_2_/N_2_ (v/v) at elevated temperatures and pressures. The high resolution XPS results indicated that the F/C ratio was 17.8% and 5.61% for Hummers GO and Staudenmaier GO, respectively.

Despite the high activity, the fluorination of graphene using F_2_ is limited by poor controllability of the C–F bonding characters (semi‐ionic or ionic bonds) and F/C ratios, special equipment requirements and environmental hazards (high toxicity and corrosion). Thus, many other fluorine‐containing agents such as SF_6_, SF_4_ or MoF_6_ were also used for fluorinating graphene. Pumera et al.[[qv: 22d]] demonstrated the fluorination of GO using SF_6_, SF_4_ or MoF_6_. The surface elemental composition showed that GO synthesized by the Hummers method were thermally fluorinated using SF_6_, SF_4_ and MoF_6_ at 800 °C with different F/C ratios of 1.92%, 0.53%, and 0.26%, respectively. Additionly, GO synthesized by Staudenmaier method were treated by microwave in SF_6_ at 800 °C and 1000 °C and showed diffeirent F/C ratios of 4.25% and 0.49%, respectively. The results revealed that F/C ratios of GO could be tuned by different gaseous fluorine‐containing agents with the control of the temperature for the fluorination. The structural changes of GO also led to the changes of F/C ratios under the treatment of SF_6_.[[qv: 22d]] Despite recent progress, the fluorination using XeF_2_, SF_6_, SF_4_ or MoF_6_ is still far from up‐scale industrial production. Thus, exploring a low‐toxic fluorine‐containing gas (or mixed gas) for mild, selective and high efficient fluorination is important for preparing various fluorianted graphene in the future.

#### 2.1.2 Plasma Fluorination

**Figure 3:**
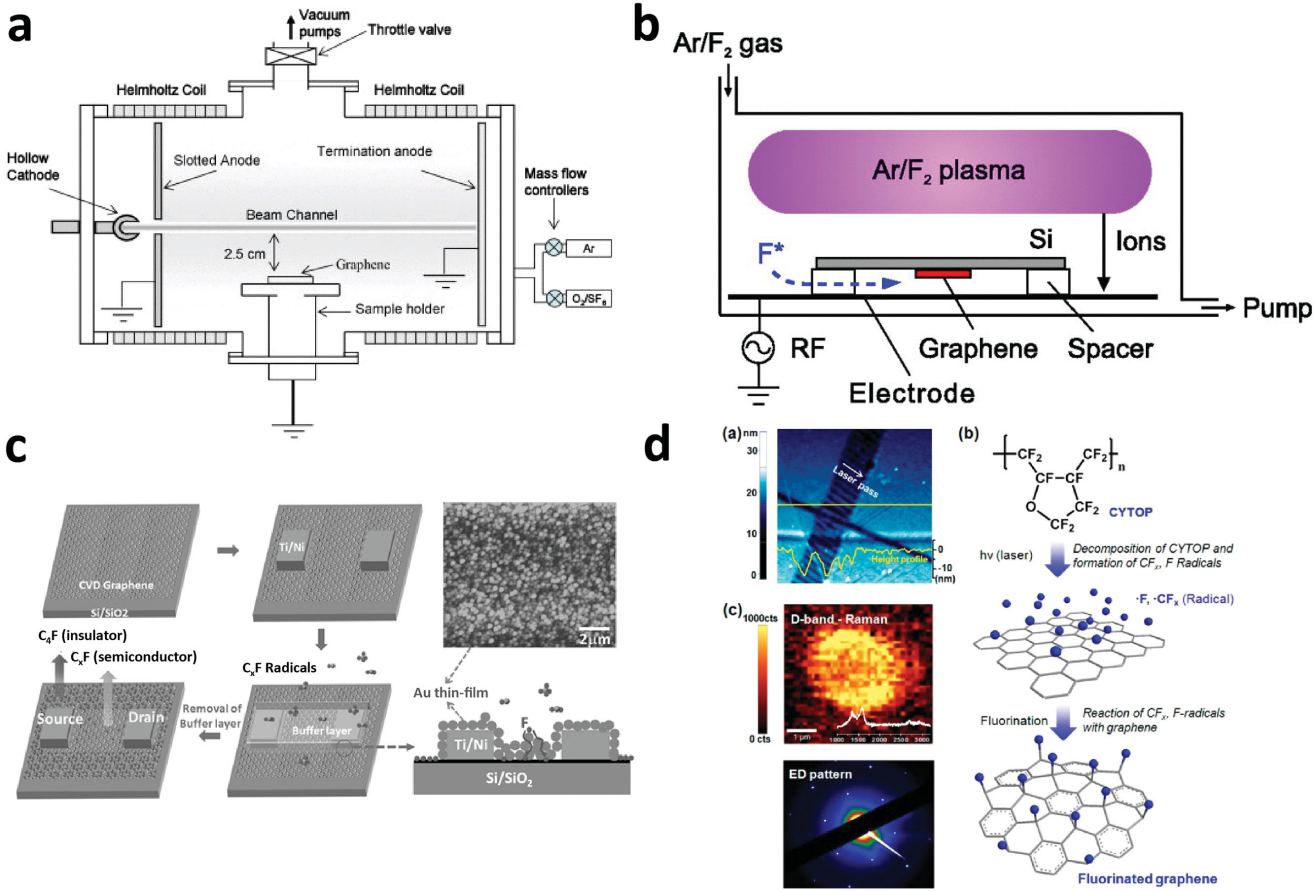
a) Schematic diagram of the plasma processing systems in Ar/SF_6_ mixtures. Reproduced with permission.[[qv: 23a]] Copyright 2010, American Institute of Physics. b) Schematic view of reactive ion etching system used for fluorination of grapheme in Ar/F_2_ plasma. Reproduced with permission.[[qv: 23h]] Copyright 2012, American Institute of Physics. c) Schematic of the one‐step formation of a transistor by using a patterned buffer layer during the CF_4_ plasma treatment. Reproduced with permission.[[qv: 23e]] d) The scheme showing a mechanism for fluorination by using CYTOP and laser irradiation. Reproduced with permission.[[qv: 25a]] Copyright 2012, American Chemical Society.

Compared with severe fluorination of fluorine‐based gas, plasma fluorination is considered to be an easy to control, mild and clean method for preparing fluorinated graphene. During plasma fluorination, the fluorine radicals generated by the plasma technique adsorb onto graphene and form different C‐F bonds. Recently, a variety of plasma sources, such as SF_6_,[[qv: 23a,c,f,g,j]] CF_4_,[[qv: 23b,d,e,i,k]] and F_2_,[[qv: 23h]] have been used. Baraket et al.[[qv: 23a]] synthesized fluorinated graphene using electron‐beam generated plasmas in Ar/SF_6_ (**Figure** **3**a), and found that C‐F bonds in fluorinated graphene could be reduced to original C–C bonds by removing F atoms via annealing (500 °C). Sherpa et al.[[qv: 23g]] reported that the polarity of C‐F bonds, depending on the C‐F bonding characters (ionic, semi‐ionic, or covalent) between F and C atoms, could be induced in fluorinated epitaxial graphene using a SF_6_ plasma‐treatment in a reactive ion etcher system. They found that work function of fluorinated graphene was controlled by the polarity of C‐F bonds as well as by the degree of fluorination. Recently, plasma fluorination of graphene using SF_6_ plasma was also investigated by Yang et al.[[qv: 23j]] Interestingly, the fluorination of single‐layer graphene is much more feasible than multi‐layer because of large corrugations.

Bon et al.[[qv: 23b]] reported the fluorination of GO, obtained from thermally exfoliated graphite oxide, by the treatment of CF_4_ plasma. C‐F bonds in fluorinated graphene could be changed to C‐N bonds by reacting with butylamine (the nucleophilic reagent) at room temperature.[[qv: 23b]] Yu et al.[[qv: 23k]] also synthesized fluorinated reduced graphene oxide (RGO) using CF_4_ plasma at room temperature, and the F/C ratios (F/C ≈ 0.17–0.27) were controlled by the plasma exposure time. Recently, Wang et al.[[qv: 23i]] reported the fluorination of CVD‐grown single‐layer graphene using CF_4_ plasma. The results showed that F/C ratios of fluorinated graphene were tuned by the conditions of the plasma; however, the resultant fluorinated graphene and fluorographene consisted of a mixture of CF_x_ (x ≈ 1–3), and the spatial distribution of F on graphene was highly inhomogeneous.[[qv: 23i]] K. I. Ho et al.[[qv: 23e]] presented a one‐step approach for the selective fluorination of graphene using CF_4_ plasma in a plasma‐enhanced chemical vapor deposition (PECVD) system. During the fluorination, F‐radicals preferentially fluorinated graphene at a low temperature (<200 °C), while the defect was suppressed by screening out the effect of ion damage (Figure 3c). When the fluorination time increases, D peak in pristine graphene is remarkably intensified and the G peak is broadened. Simultaneously, the D' peak originated from the intra‐valley resonance of Raman Scattering, is obvious.[[qv: 23e]]

In addition to SF_6_ and CF_4_, F_2_ is also used for plasma fluorination. Tahara et al.[[qv: 23h]] developed a highly controlled fluorination method of preparing fluorinated graphene utilizing fluorine radicals in Ar/F_2_ plasma. To overcome ion attacks and facilitate the C=C addition reaction of graphene with fluorine radicals, graphene was placed on the other side of the Si substrate to avoid direct contact with Ar/F_2_ plasma (Figure 3b).

High‐density plasma is important for fluorinating graphene with high F/C ratios because the fluoride‐containing ions (such as F^−^, CF_4_^+^, CF_3_^+^) energies are lower than fluoride radicals. Desipte a simple and effective method, the plasma fluorination inevitably damages the carbon structure of graphene by severe ion bombardment at a relatively high temperature.[[qv: 25a]],^28^ Furthermore, the production is limited because the preparation is highly restricted to plamas‐treated area and expensive equipment. And ion damage during the plasma treatment is inevitable. Thus, the up‐scale production of fluorinated graphene via plasma fluorination needs more developed technique and equipments.

#### 2.1.3 Hydrothermal Fluorination

Hydrothermal or solvothermal fluorination is another versatile method for preparing fluorinated graphene and fluorographene. The fluorination effect depends on fluorine precursors, such as hydrofluoric acid (HF),[[qv: 24c]] BF_3_‐etherate[[qv: 24b]] and diethylaminosulfur trifluoride (DAST)[[qv: 24a,e]] and hexafluorophosphoric acid (HPF_6_).[[qv: 24d]]

Wang et al.[[qv: 24c]] presented a convenient method to fluorinate dispersed GO using HF through a simple hydrothermal process. Note that some oxygen‐containing groups were substituted by F atoms during the hydrothermal reaction. In addition, the F/C ratios were controlled by varying the temperature, times and HF concentration. Similarly, Gao et al.[[qv: 24a]] reported the solvothermal fluorination of GO films through converting the oxygen‐containing groups (mainly hydroxyl, epoxy, and carbonyl/carboxylic) to C‐F bonds by treating GO with DAST in chloroform at 50 °C. More recently, Samanta et al.[[qv: 24b]] prepared fluorinated RGO with fluorine coverage of 38 wt% using anhydrous BF_3_‐etherate and alkyl thiol/alkyl amine on the gram scale.

GO is an excellent nanosheet for the hydrothermal fluorianation because of many epoxide, hydroxyl, carboxylic and ketone functional groups on the surface The oxygen‐containing groups can be removed or substituted by the formation of C‐F bonds at high temperature using a suitable fluorination solvent. Thus, the hydrothermal fluorination shows great potential for fluoriated graphene with high F/C ratios. Unfortunately, the uniform distribution of C‐F bonds on fluorinated graphene by the hydrothermal fluorination has yet been reported.

#### 2.1.4 Photochemical/Electrochemical Synthesis

Lee et al.[[qv: 25a]] reported an environmentally friendly method of selectively fluorinating single‐side graphene using a solid fluoropolymer CYTOP (Cytop, CTL‐809) source and laser irradiation. The fluoropolymer CYTOP decomposed under laser irradiation on the surface of a single‐layer graphene film on a SiO_2_/Si substrate. Active fluorine radicals, generated by the decomposition of CYTOP, reacted with the sp^2^‐hybridized carbon and formed C‐F bonds (Figure 3d). Gong et al.[[qv: 25c]] prepared fluorinated RGO by employing UV irradiation on GO dispersion in HF at room temperature. The synthesis of oxy‐fluorinated graphene via an electrochemical method was demonstrated by Bruna et al.[[qv: 25b]] A graphite flake contacted a platinum wire as the working electrode was fluorinated in HF (50 wt%) as the electrolyte. Despite an environmentally friendly method, the F/C ratios of fluorinated graphene by photochemical fluorination are relatively low, and the special fluorination agents have yet to be developed.

### 2.2 Exfoliation Methods

#### 2.2.1 Sonochemical Exfoliation

Sonochemical exfoliation of multilayer materials has been well researched because it is a versatile and nondestructive technique for preparing high‐quality two‐dimensional single‐ or few‐layer nanomaterials. Solution‐processed exfoliation has been employed for up‐scale production of two‐dimensional graphene and MoS_2_.^29^ Single‐layer fluorinated graphene and fluorographene were obtained by exfoliation from fluorinated graphite assisted by ultrasonication. To date, many intercalated molecules have been used to exfoliate fluorinated graphene including sulfolane,[[qv: 9a]] ionic liquids,[[qv: 26a]] surfactant,[[qv: 26d]] N‐methyl‐2‐pyrrolidone (NMP),[[qv: 26b]],^30^ chloroform,[[qv: 26g]] 2‐isopropanol (IPA)[[qv: 26e]] and acetonitrile.[[qv: 26c]] The driving force of the intercalation can be evaluated by Gibbs free energy (Δ*G*) of the intercalation compounding process triggered by the F atoms, which is defined in Equation (1)

(1) ΔG=ΔH−TΔS

where Δ*H* and Δ*S* are the enthalpy and entropy for the intercalation of molecules or solvents respectively. Because of van der Waals attraction between two adjacent layers of fluorinated graphite, Δ*H* is generally expected to be positive; resulting in a small and positive *ΔG*, and thus the exfoliation is mainly affected by *T*Δ*S*. At high temperature and pressure, the increase in Δ*S* leads to a decrease in Δ*G*, which indicates increasing driving forces. Thus, compared with graphite, fluorinated graphene is easily exfoliated by the intercalation of molecules with relatively weak van der Waals attraction and a large interlayer space. High‐yield single‐ or few‐layer fluorinated graphene with a specific F/C ratio is obtained.

**Figure 4:**
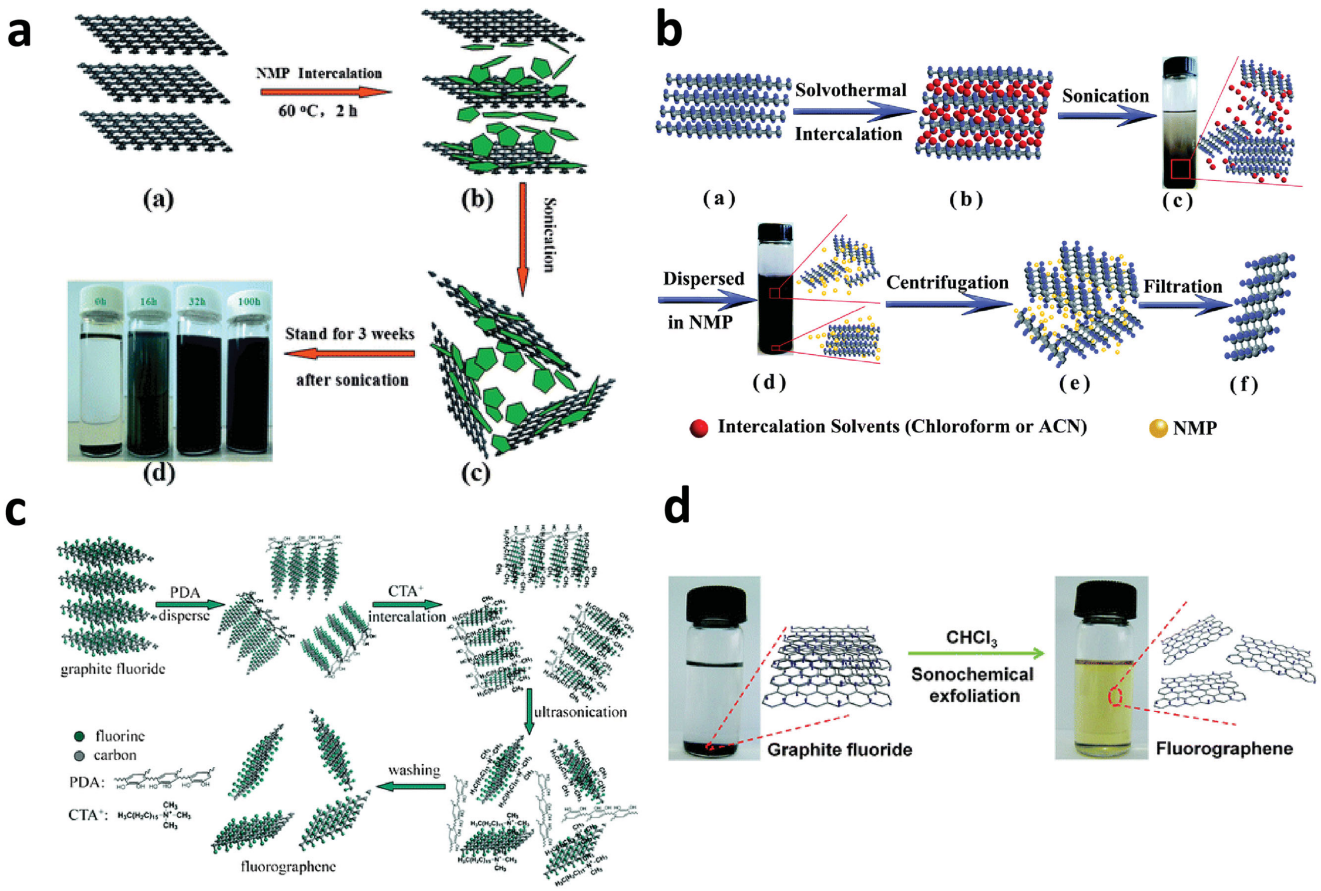
a) Schematic of the NMP intercalation and exfoliation fabrication processes for fluorographene dispersions.Reproduced with permission.[[qv: 26b]] Copyright 2012, Royal Society of Chemistry. b) Schematic of the preparation of fluorographene nanosheets by the solvothermal intercalation and exfoliation. Reproduced with permission.[[qv: 26c]] Copyright 2014, Royal Society of Chemistry. c) Schematic of the cooperative exfoliation process by PDA and CTAB to prepare fluorinated graphene sheets. Reproduced with permission.[[qv: 26d]] Copyright 2012, Royal Society of Chemistry. d) Illustration of the fabrication of fluorographene sheets via a chloroform‐mediated sonochemical exfoliation method. Reproduced with permission.[[qv: 26g]] Copyright 2013, Royal Society of Chemistry.

Zborˇil et al.[[qv: 9a]] prepared fluorinated graphene (F/C = 1.00) by a single‐step liquid‐phase exfoliation. In this process, fluorinated graphene was exfoliated from commercial fluorinated graphite suspended in sulfolane at a 135 W ultrasonic bath for 1 h at 50 °C. Chang et al.[[qv: 26a]] reported an effective and low‐cost exfoliation to obtain single and few‐layer fluorinated graphene (F/C = 0.25 or 0.50) in ionic liquid. In this method, ionic liquid (1‐butyl‐3‐methylimidazolium bromide) intercalated into the interlayer of commercial fluorinated graphite by mixing and incubating. After intercalation, black colloidal dispersion of fluorinated graphene was obtained by ultrasonication. Transmission electron microscopy (TEM) and atomic force microscopy (AFM) images revealed that two‐dimensional fluorinated graphene showed 1–5 layers with 2–10 μm in edge size. Among many organic solvents, NMP is considered to be an important intercalated molecule to exfoliate fluorinated graphene because of its dipole moment value of 4.09 D (**Figure** **4**a).[[qv: 26b]] According to Gong's studies, the intercalation of NMP into the interlayer of fluorinated graphite was accomplished by refluxing for 2 h, and the subsequent exfoliation was facilitated by ultrasonication for 100 h. Feng et al.[[qv: 26c]] reported a solvothermal exfoliation to prepare few‐layer (1–3) fluorographene with a high‐yield production of 15%. The semi‐ionic C‐F bonds of fluorographene exfoliated by chloroform (Figure 4b) might be a result of hydrogen bonding during the intercalation. In addition, Wang et al.[[qv: 26d]] prepared fluorinated graphene by exfoliation of fluorinated graphite using a cationic surfactant of cetyl‐trimethyl‐ammonium bromide (CTAB) and dopamine (DA). This intercalation was carried out at room temperature in air (Figure 4c). Zhu et al.[[qv: 26g]] demonstrated an easy method to synthesize fluorinated graphene nanosheets by means of a one‐pot sonochemical exfoliation of the commercially available graphite fluoride powders in chloroform under ambient conditions without any additional pretreatments, assistant reagents, or special protections (Figure 4d).

Liquid‐phase exfoliation is a relatively simple method for high‐quality fluorinated graphene by optimizing the intercalation. The exfoliation at room temperature could reserve most original fluorine atoms. The chemicals for the intercalation and the reaction condition (time, temperature and pressure) are significantly important for the liquid‐phase exfoliation. In general, polar molecules are more effective for the intercalation than the non‐polar molecules. Besides, high temperature, long‐term and high pressure facilitate the exfoliation for single‐ or few‐layer fluorinated graphene after ultrasonication and separation. However, C‐F bonds of fluorinated graphene might be partially reduced during the high‐temperature exfoliation, and importantly, the numer of layers is hardly controlled because of the weak selectivity of the exfoliation.

#### 2.2.2 Modified Hummer's Exfoliation

Hummer's method was widely used to prepare GO by the intercalation and oxidation of bulk graphite. Recently, the modified Hummer's method attracted tremendous attention to the exfoliation of fluorinated graphite because of its convenient, easy‐control process.^20^,[[qv: 21b]],^31^ Pulickel M. Ajayan et al.[[qv: 21b]],[[qv: 31c]] developed a methodology to synthesize fluorinated GO using a modified Hummer's method. The magic‐angle spinning (MAS) 13C NMR results revealed that there were two types of fluorinated GO: partially fluorinated GO (FGO) and highly fluorinated GO (HFGO). FGO was hydrophilic similar to GO in hydrophilicity, while HFGO was relatively hydrophobic. Although the modified Hummer's method improves the dispersion of fluorinated GO in water or organic solvents by introducing numerous oxygen‐containing groups, this severe reaction inevitably partially destroys C‐F bonds of fluorinated graphene.

#### 2.2.3 Thermal Exfoliation

Fluorographene can also be exfoliated from fluorinated graphite by thermal exfoliation. Dubois et al.^27^ prepared fluorographene by thermal exfoliation of fluorinated HOPG prepared using F_2_. Fluorographene was obtained by fast elimination of interlaminar species of fluorinated HOPG with a sharp increase in temperature accompanied by the color changing from greyish to black.^27^

## 3 Structures

C‐F bonding character including C‐F bonds, F/C ratio, and configuration largely determines the chemical (electrochemical), electrical, electronic, optical, magnetic structures, stability and hydrophobicity of fluorinated graphene. Thus, the deep understanding of fluoro‐carbon structure is fundamental to control the properties and design the application of fluorinated graphene. In this section, we discusse the C‐F structural characteristics controlled by a variety of methods or technologies to offer a strategy for tuning C‐F bonds precisely and uniformly.

### 3.1 C‐F Bond

**Figure 5:**
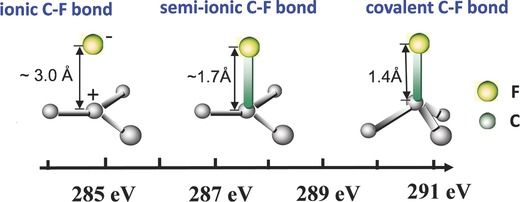
The length of C‐F bons and the characteristic peaks of C‐F bond in C1s XPS.

Chemical bonds are usually determined by the electronegativity between two bonding atoms. As a result, C‐F bonds vary from covalent bonds, through semi‐ionic bonds, to ionic bonds because of the extremely high electronegativity of fluorine. This feature results in a more electrostatic character in the covalent C‐F bond.[[qv: 14b]],^32^ Sato et al.^33^ experimentally confirmed the existence of semi‐ionic C‐F bonds in fluorine‐graphite intercalation compounds. Recently, Lee et al.[[qv: 14d]] synthesized fluorinated graphene with semi‐ionic bonds through a one‐step liquid fluorination using liquid ClF_3_ as the fluorine agent. Moreover, semi‐ionic C‐F bonds in fluorine‐graphite intercalation compounds and fluorinated graphene were also reported based on theoretical calculations.^15^, ^34^ However, the length of semi‐ionic and ionic C‐F bonds has never been experimentally determined.[[qv: 14a]],[[qv: 13b]],^34^, ^35^ The semiempirical result is shown in **Figure** **5**.

The fluorination of C–C bonds of graphene usually contains two competing reaction processes: (1) fluorine radicals react with graphene to form covalent C‐F bonds, in which the sp^3^‐hybridized C atoms connect to F atoms and (2) fluorine radicals react with graphene to form semi‐ionic C‐F bonds, in which the sp^2^‐hybridized C atoms connect to F atoms. C–F bonds change from ionic to semi‐ionic to covalent, accompanied by a decrease in F/C ratios by changing the fluorination conditions (e.g., fluorination agents, temperature and time).[[qv: 14a]] Borini et al.[[qv: 25b]] reported oxy‐fluorianted graphene with semi‐ionic C‐F bonds through the electrochemical intercalation of graphite in hydrofluoric acid solution. Wang et al.[[qv: 19b]] found that C‐F bonds showed the partial transformation from semi‐ionic nature to covalence with the increasing F/C ratios of fluorinated grapehene, which was controlled by the exposure time in XeF_2_ atmosphere. This transformation was also found in the liquid‐phase exfoliation with the appropriate solvent such as chloroform. Feng et al.[[qv: 26c]] found the partial transformation of covalent to semi‐ionic C‐F bonds in fluorinated graphene exfoliated by chloroform due to the formation of C–H…F hydrogen bonds between chloroform molecules and F atoms of fluorinated graphite.[[qv: 22a]],[[qv: 33b]],^36^ Additionly, the low exfoliation temperature could contribute to the appearance of Csp_2_‐F bonds.[[qv: 33b]] Previous studies indicated that the partial transformation between ionic (semi‐inoic) and covalent bonds could be caused by the interaction between C‐F bonds and other molecules or materials. Importantly, the natrue of C‐F bonds have a significant impact on the properties of fluorinated graphene, such as work function,[[qv: 23g]] reaction activity,^37^ and electrochemical performance.^38^

The presence and percentage of covalent, semi‐ionic or ionic C‐F bonds in fluorinated graphene are investigated by X‐ray photoelectron spectroscopy (XPS) and Fourier transform infrared spectroscopy (FTIR). According to previous studies, the characteristic peaks of semi‐ionic bonds between C and F atoms were observed at approximately 287–290 eV in the C1s XPS spectra (Figure 5), 685–688 eV in the F1s XPS spectra and 1050–1150 cm^−1^ in the FTIR spectra.[[qv: 19b]],[[qv: 22a]],[[qv: 25c]],[[qv: 26c,f]],^39^ The characteristic peaks of C‐F bonds in fluorinated graphene are given in **Table** **3**. Unfortunately, fluorinated graphene containing ionic C‐F bonds has seldom been reported.

**Table 3:** Comparison of C‐F bonds in fluorinated graphene synthesized with different methods

Method	Raw materials		Peak location in FTIR (cm^−1^)		Peak location in XPS (eV)		Ref.
	Graphene‐based materials	Fluorine agents/exfoliation solvents	Covalent C‐F	Semi‐ionic C‐F	Covalent C‐F	Semi‐ionic C‐F	
Direct gas‐fluorination	CVD graphene	XeF_2_	1211	1112	F1s 687.5	F1s 685.5	[[qv: 19b]]
Direct gas‐fluorination	GO	F_2_	1221	1150	C1s 289.7	C1s 288.0	[[qv: 22a]]
Direct gas‐fluorination	RGO	F_2_/N_2_ mixed gas	1212	1113	C1s 289.3	C1s 288.5	[[qv: 62b]]
Photochemical synthesis	GO	HF solution	1212	1149	C1s 291.2	C1s 290.3	[[qv: 25c]]
Hydrothermal fluorination	Graphene	ClF_3_	1225	1107–1120	C1s 289.4	C1s 288.1	
					F1s 689.6	F1s 686.8	
Sonochemical exfoliation	Fluorographite	NMP	1212	1084	C1s 290.8	—	[[qv: 26f]]
Sonochemical exfoliation	Fluorographite	Cloroform	1216	1143	C1s 289.9	C1s 288.4	[[qv: 26c]]
					F1s 689.0	F1s 688.1	[[qv: 39a]]
Thermal exfoliation	FGO	—	—		C1s 290.3	C1s 288.4	[[qv: 39b]]

### 3.2 F/C Ratio

Precise control of the F/C ratio of fluorinated graphene is important for opening the bandgap, tuning electrical conductivity and optical transparency and understanding the structural transformation. Thus, beyond the aforementioned techniques in the second section, fluorination conditions, including the reaction temperature, the species of fluorination agents and catalysts, the type of carbon (e.g., graphene, GO, and RGO), the treated side and the sonochemical time, are utilized to tune the F/C ratios of fluorinated graphene.[[qv: 23e,k]],[[qv: 24c]],[[qv: 26b]],^40^

Yu et al.[[qv: 23k]] reported that F/C ratios (0.17–0.27) of fluorinated graphene were controlled by the time of CF_4_‐plasma treatment. Similar results were also observed in a recent study by Kuan‐I Ho et al.[[qv: 23e]] In addition, Wang et al.[[qv: 24c]] presented an easy, low‐cost and efficient hydrothermal‐process to tune F/C ratios of fluorinated graphene. The contents of each C‐F‐containing group (such as C–CF, C–CF_2_, and CF–CF_2_, CF, CF_2_, and CF_3_) were dependent on the reaction temperature, time, and HF amount. An increase in the F/C ratios (from 0.11 to 0.48) was mainly attributed to the formation of the CF–CF_2_ group.[[qv: 24c]] Interestingly, Robinson et al. found that fluorine saturation coverage differed when graphene films were fluorinated by XeF_2_ on one or both sides. X‐ray photoelectron spectroscopy and Raman spectroscopy revealed that fluorine coverage saturates at 25% (C_4_F) for one‐side fluorination and at 100% (CF) for double‐side fluorination in XeF_2_ at room temperature.[[qv: 12a]] Gong et al.[[qv: 26b]] also reported that the F/C ratios decreased with increasing ultrasonication time in NMP, which might be attributed to the increasing stretching vibration energy of C–F groups gained from the sonic power facilitating the departure of fluorine.[[qv: 26b]]

### 3.3 Configuration

**Figure 6:**
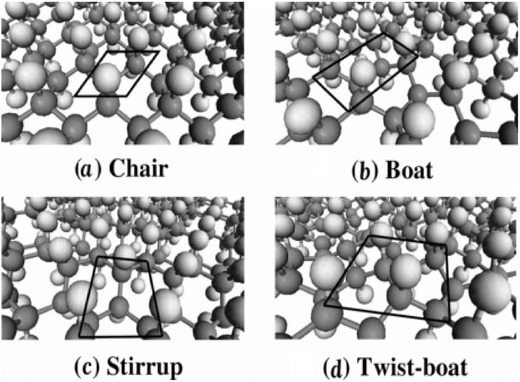
Perspective views of optimized ordered configurations of fluorographe: a) chair, b) boat, c) stirrup, and d) twist‐boat, respectively. Light and dark grey spheres represent F and C atoms, respectively. The black box indicates the unit cell employed in the calculations. Reproduced with permission.[[qv: 12b]]

Fluorinated graphene and fluorographene consisting of weakly bound stacked two‐dimensional carbon monofluorides are a basic building block of fluorinated graphite.[[qv: 4a]],[[qv: 12a]],^41^ To gain insight into C‐F bonds, theoretical calculation on the configuration of fluorinated graphene is studied, such as chair, boat, stirrup, and twist‐boat configuration (**Figure** **6**).[[qv: 12b]] The chair configuration shows a two‐dimensional alternate layer of F atoms and C atoms on both sides, whereas in a boat configuration, F atoms alternate with C atoms in pairs.[[qv: 12b]],^42^ In the stirrup configuration, each C atom is bonded to an F atom in the way that consecutive fluorine layers along a zigzag direction alternate with graphene layers, while the twist‐boat configuration derived from the boat configuration has a slight twist to F atoms connecting two unique C atoms.^43^ Different configurations of fluorographene results in different properties including binding energy, chemical activity, stability, bandgap, Young's modulus and the lattice constant.[[qv: 12b]],[[qv: 43a]] For example, fluorinated graphene or fluorographene with the chair configuration has a lower theoretical binding energy than any other configuration, and the stirrup configuration is more stable than the boat and twist‐boat configurations.[[qv: 12b]],[[qv: 43a]],^44^

The first‐principles density functional theory (DFT) calculation showed that fluorographene with the chair configuration had a direct bandgap of 3.1 eV,^45^ which is in good agreement with the experimental data, while the calculated data (7.4 eV) based on the GW (where GW refers to the one‐particle Green's function with the dynamic screened Coulomb interaction) approximation (7.4 eV) was twice as large as the experimental values.[[qv: 4a]],[[qv: 12b]],[[qv: 43a]] There are different bandgap values between DFT and GW because GW full account of the quasiparticles and their interaction with light in fluorographene included electron–hole (e–h) and electron–electron (e–e) interactions.[[qv: 12b]] The high Young's modulus E of the chair configuration was up to ≈228 N m^−1^, which was twice the experimental value (100 ± 30 N m^−1^).[[qv: 4a]] The difference between the calculation and experiment might be attributed to a large number of structural defects in fluorographene because a certain portion of C atoms was not bonded to F atoms but formed dangling bonds.[[qv: 43a]] However, compared with fluorinated carbon nanotubes, the energy difference among various configurations of fluorographene is very small, and this result indicates that fluorographene is unlikely to be a pure single‐crystal form in a chair, stirrup, boat, or twist‐boat configuration.

## 4 Properties

Fluorinated graphene shows many excellent properites such as wide bandgap of 3.1 eV, the highest theoretical specific capacity (865 mA h g^−1^), good thermal stability below 400 °C, distinct nonlinear feature and high hydrophobicity. In this section, we discuss a variety of properties including bandgap, absorption or luminescence, stability, electronic conductivity, dspersibility, magnetic properties, tribological properties, mechanical or micromechanical properties, and thermal conductivity. These properties are significantly important for the application of fluorinated graphene.

### 4.1 Band Gap

Graphene shows great potential for advanced electronic devices because of unique electronic properties, such as zero bandgap and high carrier mobility up to 200 000 cm^2^ V^−1^ s^−1^.^41^, ^46^ However, a zero band‐gap, specifically valence (π) and conduction band (π*) touching at a Dirac point, lowers achievable on‐off ratios for field emission transistors based on a graphene semiconductor.[[qv: 3b]],^47^ Thus, opening the bandgap is crucial for the design and fabrication of high‐performance graphene‐based electronic devices. Theoretically, fluorographene shows a wide bandgap of 3.1 eV because of the transformation from the trigonal sp^2^ orbital to the tetragonal sp^3^ orbital.[[qv: 4a]],[[qv: 9a]],^12^, ^48^ This feature offers great potential for tuning the bandgap of fluorographene with different C‐F bonding characters.

**Figure 7:**
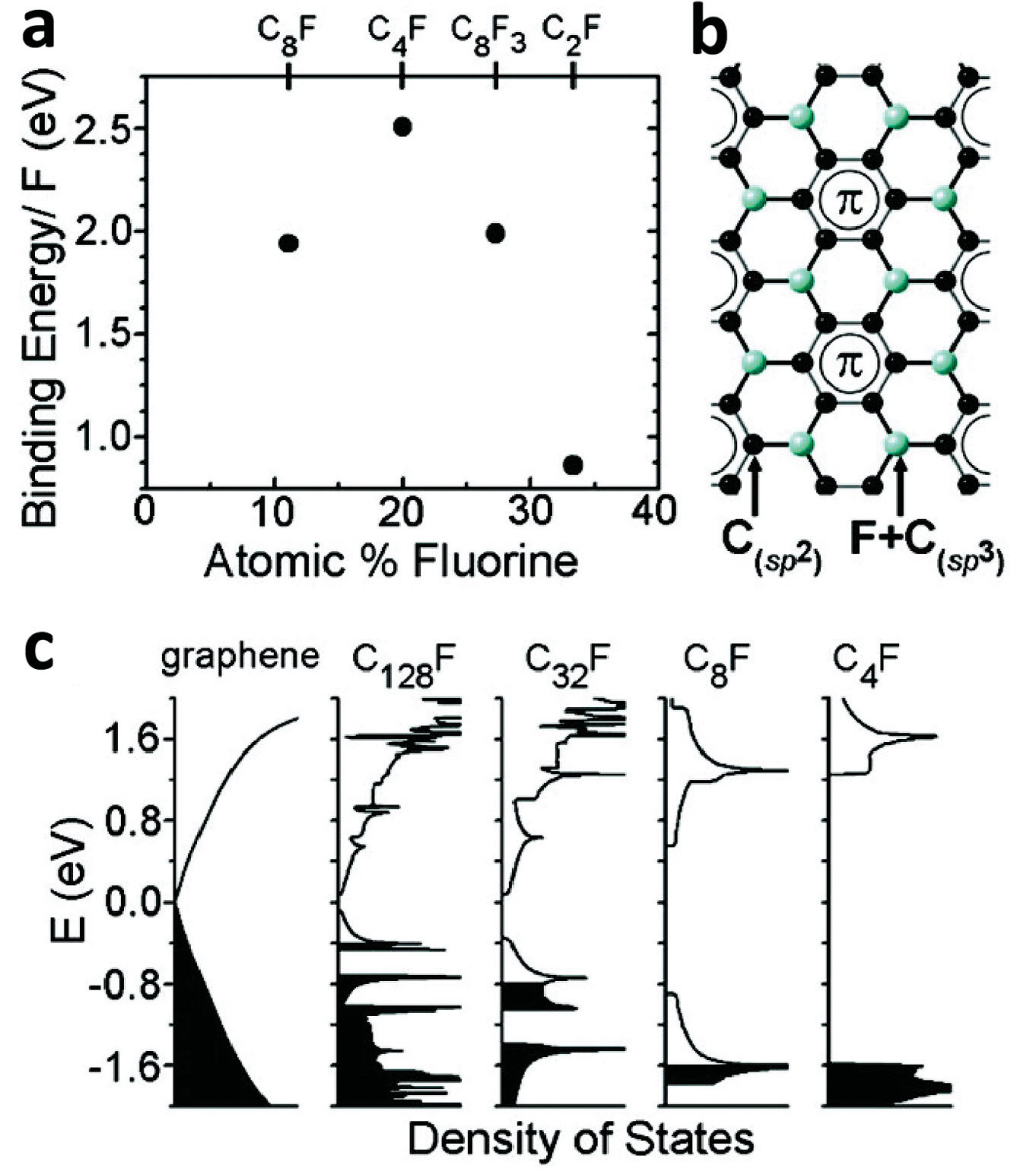
a) Calculated binding energy per F atom compared to the F_2_ gas state. b) Sketch of the calculated C_4_F configuration for the 25% coverage from (a). c) Calculated total density of states of single‐side fluorinated graphene for several fluorine coverages. Reproduced with permission.[[qv: 12a]] Copyright 2010, American Chemical Society.

Robinson et al.[[qv: 12a]] prepared fluorinated graphene films (on one side) with fluorine coverage of 25% (C_4_F) using XeF_2_. The calculation indicated that the bandgap of fluorinated graphene increases with an increasing F/C ratio because of the interaction between the p‐orbital of F and the π‐orbital of C. The formation of sp^3^ bonds led to a large change in charge densities and scattering centers in the conduction band (**Figure** **7**).[[qv: 12a]] The band gap of C_4_F is 2.93 eV according to the density of states calculations. Moreover, when graphene films were fluorinated on both sides, fluorographene (C_1.0_F_1.0_) showed a large bandgap of 3.07 eV.[[qv: 12a]] Liu et al.^48^ investigated the bandgap of fluorinated graphene with different F/C ratios. The results indicated that the C‐F bonds in low‐fluorine‐coverage fluorinated graphene (CF_0.031_, CF_0.056_, and CF_0.125_) were polar covalent bonds because of the high electronegativity of F atoms, and thus, they exhibited a metallic behavior. This behavior could be changed by increasing F/C ratios. CF_0.25_ and fluorinated graphene (CF_1.0_) had wide bandgaps of 2.92 eV and 3.13 eV, respectively, according to the generalized gradient approximation (GGA) calculations. Interestingly, fluorinated graphene (CF_0.5_) in which C atoms bonded to F atoms on one side also exhibited metallic behavior ascribed to the exchange splitting of the dangling C‐p_z_ orbital with a coupling with an impurity state induced by F atoms. The results indicate that the bandgap of fluorinated graphene is greatly influenced by F/C ratios.^48^

Based on the density‐functional GGA calculation, the bandgap can also be controlled by different configurations and layers of fluorinated graphene and fluorographene. Specifically, a chair configuration shows a bandgap of 3.10 eV, while the bandgaps of the stirrup, boat, and twist‐boat configuration are 3.58, 3.28, and 3.05 eV, respectively.[[qv: 12b]] In the chair configuration, F atoms are alternately distributed on the plane. One F atom locates above the carbon layer, while the other one is under the same layer. Thus, the chair configuration has more symmetry than the stirrup configuration. In addition, the stirrup configuration is more significant for the conduction state because charge density follows the chain characteristic.[[qv: 12b]] Li et al.^49^ calculated the bandgap of C_4_F with different layers by means of DFT computation. The results implied that bi‐layer fluorinated graphene (C_4_F) had a much narrower indirect bandgap than that of monolayer fluorinated graphene. Additionally, the bandgap of C_4_F nanosheets was further decreased by increasing the number of stacked layers because the conversion from insulator to semiconductor based on the dipole‐dipole interaction between two C_4_F layers induce a subtle interlayer polarization.^49^

### 4.2 Optical Properties

Fluorine‐substitution on carbon atom dramatically changes the optical properties of graphene including the absorption band, photoluminescence and transparency. Robinson et al.[[qv: 12a]] found that graphene film was optically transparent in the visible region after treatment by XeF_2_. The absorption coefficient decreased after fluorination by SF_6_[[qv: 23c]] and CF_4_ plasma,[[qv: 23e]] which was in agreement with other fluorinated carbon materials.[[qv: 14b]],^50^

**Figure 8:**
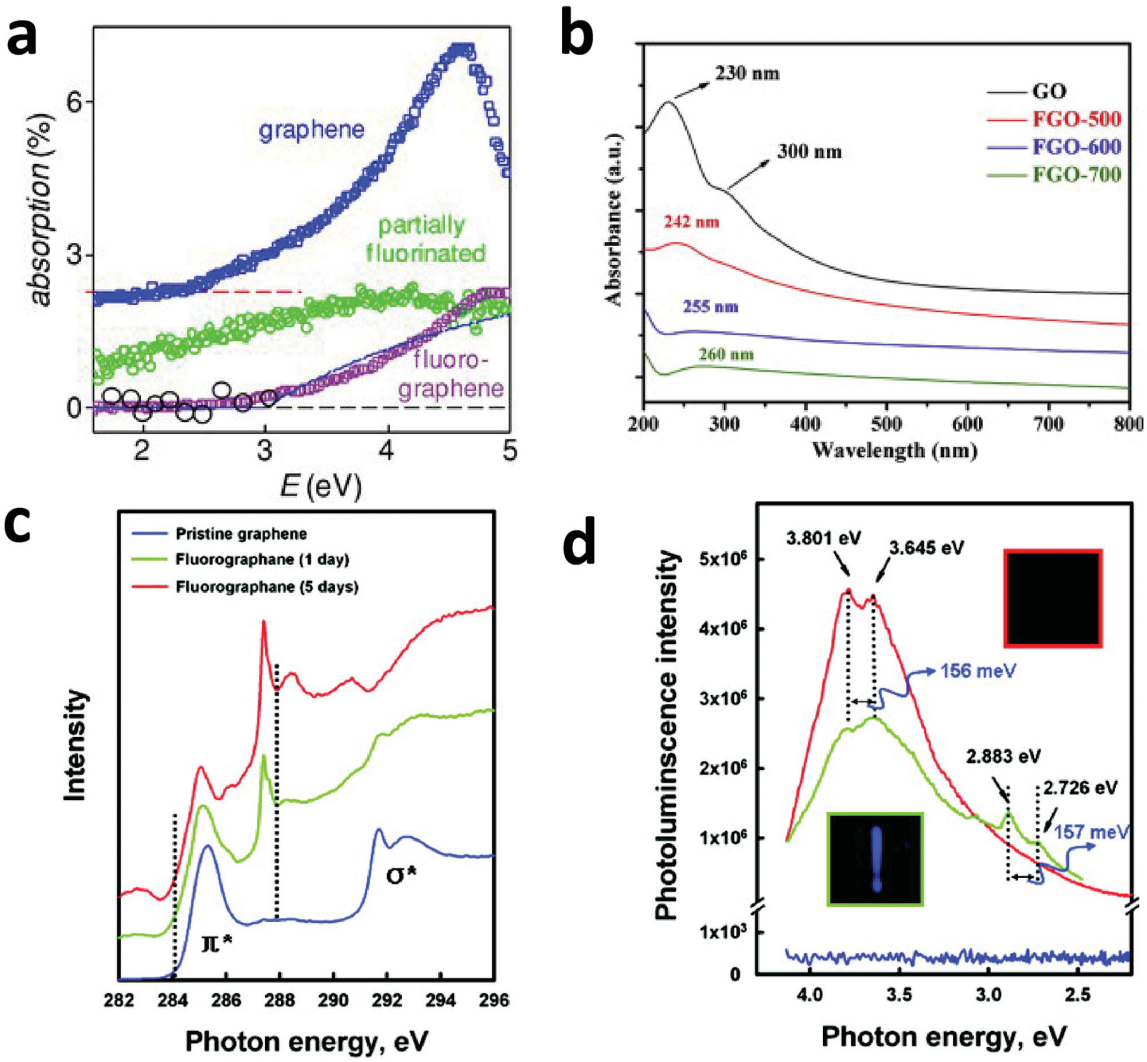
a) Changes in optical transparency of graphene due to fluorination. The absorption spectra of graphene (upper curve), partially fluorinated graphene (middle curve), and fluorographene (bottom curve). The solid curve is the absorption behavior expected for a 2D semiconductor with *E*_g_ = 3 eV. Reproduced with permission.[[qv: 4a]] b) UV–vis absorption spectra of GO (dispersed in water) and FGO (dispersed in a mixture of ethanol and NMP) just after sonication. Reproduced with permission.[[qv: 39b]] Copyright 2014, Royal Society of Chemistry. c) NEXAFS spectra of pristine graphene and fluorographene with two different contents of fluorine.[[qv: 13a]] d) Room temperature photoluminescence emission of the pristine graphene and fluorographene dispersed in acetone using 290 nm (4.275 eV) excitation. Reproduced with permission.[[qv: 13a]] Copyright 2011, American Chemical Society.

Recently, the absorption spectra of fluorinated graphene with different F/C ratios has been studied to appreciate the effect of fluorination on optical properties.[[qv: 4a]],[[qv: 24e]] Nair et al.[[qv: 4a]] investigated the optical transparency of fluorinated graphene by fluorinating in XeF_2_ at 70 °C (**Figure** **8**a). Graphene shows a peak at 4.6 eV and an absorption edge at ≈2.5 eV, which was in good agreement with a pronounced van Hove singularity, and was no longer linear above 2.5 eV (Figure 8a).[[qv: 4a]],^51^ However, the absorption spectra were drastically changed after the fluorination. Compared with graphene, fluorinated graphene showed low‐intensity absorption with a weak and broad band in the range of 4.0 to 5.0 eV. It exhibited high transparency in the whole range because of the impurity scattering.[[qv: 4a]] Furthermore, fluorographene only absorbed light with energy >3.0 eV (blue range) (Figure 8a). This result indicated that fluorographene was nearly transparent in the range of visible light with the wide bandgap ≥3.0 eV.[[qv: 4a]] Zhao et al.[[qv: 24e]] reported that fluorinated GO dispersed in CH_3_CN, synthesized by hydrothermal method with different reaction medium, exhibited two absorption peaks at approximately 220 nm and 250–350 nm, which were assigned to the π–π* transition of conjugated polyene‐type structures in the carbon nanosheets[[qv: 31b,c]],^52^ and a couple of conjugated aromatic domains with different sizes,^30^ respectively. Gong et al.[[qv: 39b]] found that the π–π* transition peak of GO red‐shifted from 230 to 260 nm after fluorination (Figure 8b) because of an increase in the π‐electron concentration and structural ordering based on the restoration of sp^2^ carbon and the possible rearrangement of atoms.[[qv: 26b]],^53^

Recently, the studies on the photoluminescence (PL) of fluorinated graphene have attracted attention because it not only yields insight into understanding electronic properties but is also crucial for advanced semiconductor devices and energy harvesting. Jeon et al.[[qv: 13a]] reported the room‐temperature PL spectra of graphene and fluorinated graphene dispersed in acetone using 290 nm (4.275 eV) excitation (Figure 8d). The results showed that fluorinated graphene (fluorination for 5 days) exhibited two emission peaks at approximately 3.80 eV and 3.65 eV indicating wide bandgaps, while no emission was obtained in graphene with zero bandgap.[[qv: 13a]],^54^ Specifically, the peak at 3.80 eV corresponded to the band‐to‐band recombination of a free electron and a hole, which was found in the bandgap of fluorinated graphene measured by near edge X‐ray absorption spectroscopy (NEXAFS) (Figure 8c).[[qv: 13a]] The peak at 3.65 eV was 156 meV (1260 cm^−1^) below the bandgap because of phonon‐assisted radiative recombination across the bandgap where the C–F vibration mode was excited when the electron‐hole pair recombined. Analogously, two accompanying peaks at 2.88 eV and 2.73 eV were also observed in low‐degree fluorinated graphene (fluorination for 1 day) (Figure 8d).[[qv: 13a]] Based on unique PL, fluorographene can be developed for fabricating flexible near ultraviolet LEDs by optimizing quantum yield.

**Figure 9:**
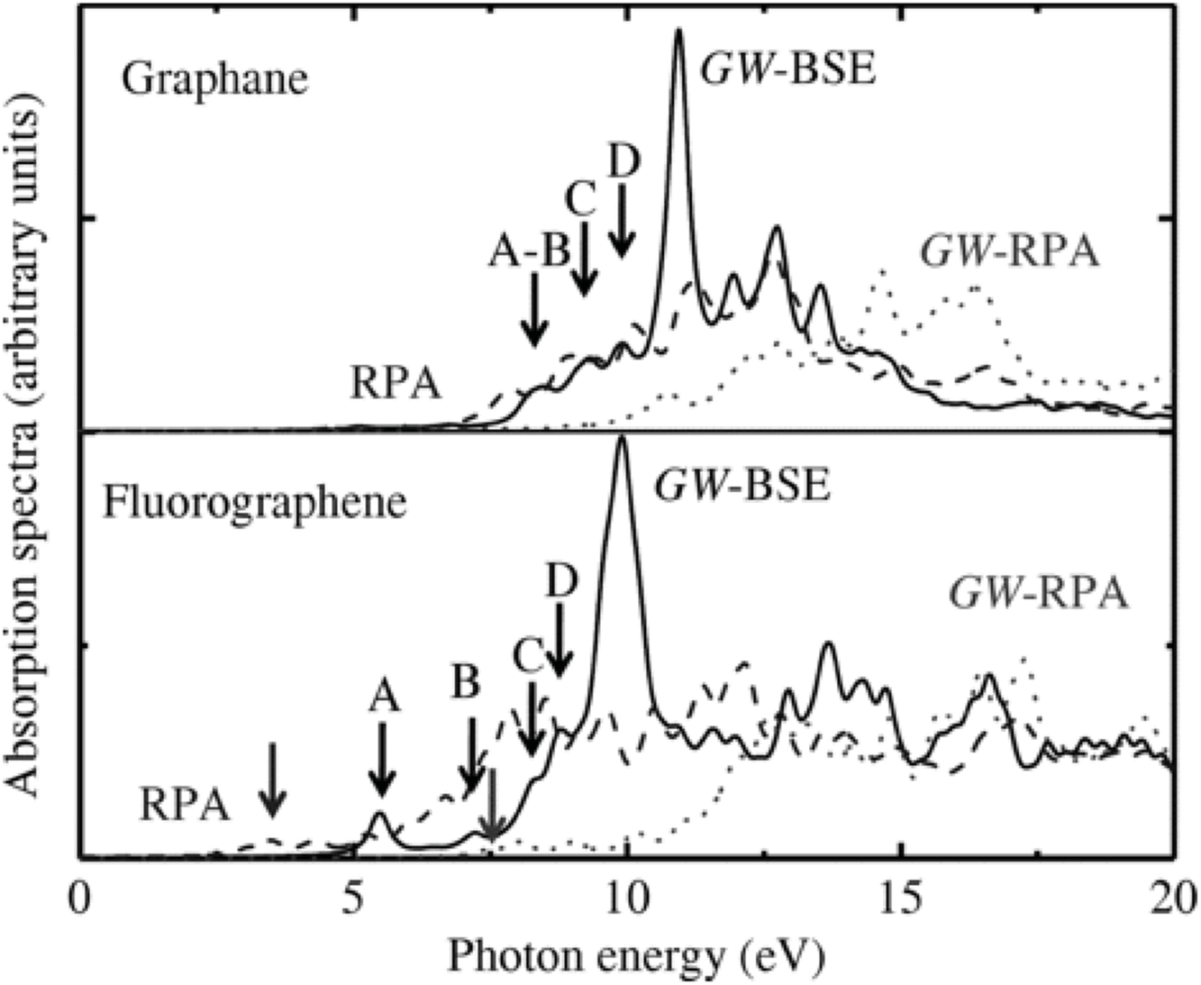
Calculated absorption spectra using RPA (dashed lines), GW‐RPA (dotted lines), and GW‐BSE (solid lines) for graphane (top panel) and flurographene (bottom panel), respectively. Reproduced with permission.[[qv: 12b]]

Optical properties of ground‐state fluorinated graphene were also predicted by theoretical calculation based on DFT.^55^ However, the calculation typically does not exactly match with experimental optical spectra because it does not take into account the interaction between two quasiparticles.^56^ In this respect, the Bethe–Salpeter equation (GW‐BSE) represents a more precise method than DFT for calculating the direct transitions because it takes into account electron–electron (e–e) and electron–hole (e–h) interaction.^[154–156]^ Samarakoon et al.[[qv: 12b]] reported the in‐plane absorption spectra of graphene and fluorographene calculated by GW‐BSE along with the random phase approximation (RPA) and GW‐RPA, respectively. RPA was regarded as the result of the DFT level. As shown in **Figure** **9**, graphene had many notable peaks around 10–12 eV as a result of strong electron–hole correlations along with the appearance of bounded excitons in the ultraviolet region, opening the path toward an excitonic Bose‐Einstein condensate in graphene that was observed experimentally.[[qv: 12b]],^56^, ^58^ This feature was also obtained for fluorographene. A distinctive peak around 9.8 eV of fluorographene emerged in GW‐BSE that was evidently connected to strong electron‐hole coupling and was attributed to the transition from the near‐gap valence bands to the minimum conduction band.[[qv: 12b]] Theoretical calculation provides an insightful understanding of optical properties controlled by C‐F bonds, and results will promote more experimental studies on quantitative and qualitative descriptions of the optical properties of fluorinated graphene in the future.

### 4.3 Stability

**Figure 10:**
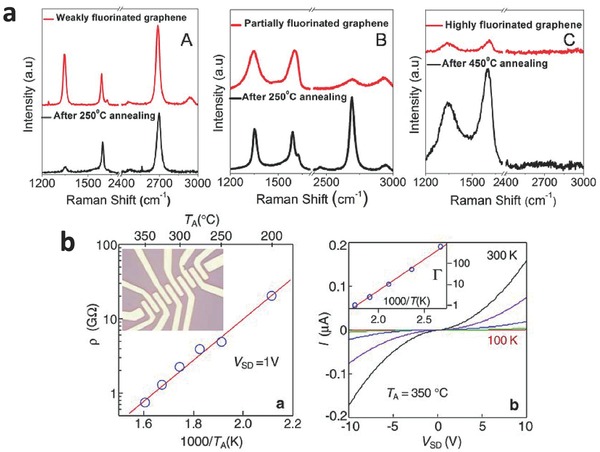
a) Raman spectra of graphene fluorinated to various levels and then annealed at different *T*. A,B,C) Raman spectra for weakly, mode rately and highly fluorinated graphene, respectively. b) Changes in fluorographene's ρ induced by annealing and *I–V* characteristics for partially fluorinated graphene obtained by reduction at 350 °C. The curves from flattest to steepest were measured at *T* = 100, 150, 200, 250, and 300 K, respectively. Reproduced with permission.[[qv: 4a]]

Compared with the instability of GO^59^ and easily decomposed graphene,^60^ fluorographene shows good chemical and thermal stability as a result of strong C‐F bonding energy.[[qv: 4a]] Raman spectroscopy (**Figure** **10**a) was typically utilized to study the stability of fluorinated graphene because it can provide a wealth of information about the structures of graphene‐based materials. The stability of fluorinated graphene with different F/C ratios at high temperature has been recently reported in several studies.[[qv: 4a]],[[qv: 12a]],[[qv: 22c]] Fluorinated graphene with a low F/C ratio could be partially recovered to pristine graphene by a short annealing‐time at temperatures <400 °C, which was reflected by a continuous decrease in the **D** band. In contrast, fluorographene shows a high stability below 400 °C .[[qv: 4a]],[[qv: 12a]],[[qv: 22c]] The removal of both C and F atoms in fluorographene was observed by a prolonged annealing time at high temperature (≈450 °C).[[qv: 4a]] In addition, according to XPS data, fluorinated graphene, prepared using XeF_2_ gas on SiO_2_, Au, and Cu substrates, lost approximately 50–80% of the initial F/C ratios over 10 days until the F/C ratios were not changed.^61^ The change in C‐F bonds by annealing at different temperatures was also demonstrated by an increase in electrical conductivity (Figure 10b).[[qv: 4a]] No current could be detected when fluorographene was annealed T_A_ below 200 °C. Fluorographene became weakly conductive, and the effective resistivity *ρ* = V/I decreased to ≈1 GΩ at 350 °C. The results indicated that the thermal stability and chemical inertness of fluorographene were similar to Teflon.[[qv: 4a]],[[qv: 13b]]

In addition, fluorographene showed a good chemical stability in many liquids such as water, acetone, and propanol, and under ambient conditions except for strong reductants.[[qv: 4a]],[[qv: 9a]] It was found that fluorinated graphene could be reduced by hydrazine, potassium iodide, ultraviolet irradiation and alkylamine compounds.[[qv: 9a]],[[qv: 12a]],^61^, ^62^ Robinson et al.[[qv: 12a]] reported the low temperature chemical reduction of fluorographene by hydrazine with the process of 4CF*_n_* + *n*N_2_H_4_ → 4C + 4*n*HF + 2*n*N_2_. Radek et al.[[qv: 9a]] provided a pathway for defluorination using KI in DMF. In this process, fluorinated graphene transformed to metastable graphene iodide, which quickly decomposed to graphene and iodine at just 150 °C: CF+ KI → KF + [CI]; [CI] → C + 1/2I_2_. Additionally, Lee and co‐workers[[qv: 14d]] reported that ionic C‐F bond was selectively reduced by acetone treatment at a low temperature with the equation 2C_2_F(semi‐ionic) + CH_3_C(O)CH_3_(l) → HF + 2C(s) + C_2_F(covalent) + CH_3_C(O)CH_2_(l).

More recently, new fluorinated graphene derivatives were prepared by the covalent modification of fluorinated graphene.^7^, ^8^, ^63^ Stine et al.[[qv: 63a]] fluorinated CVD‐grown graphene sheets followed by covalent modification with ethylenediamine. They found that the intensity of the F 1s peak was reduced by ≈90% whereas a large N 1s peak at 399.5 eV appeared because of the removal of the fluorine. Urbanová et al.^8^ synthesized thiofluorographene through the covalent functionalization (nucleophilic substitution). The thiofluorographene showed a small region where F atoms were substituted by –SH groups. Interestingly, the semiconducting properties of thiofluorographene could be potentially regulated by tuning the SH/F ratios.

### 4.4 Electronic Conductivity

Single‐layer graphene shows high electron mobility because of its sp^2^ hybridized C atoms with a p_z_ orbital forming a π*‐*conjugated bond. Fluorination is widely used to chemically tailor the electrical conducitivity because it enables the transition from metallic/semiconducting to an insulating nature controlled by different F/C ratios.

Fluorographene, the thinnest two‐dimensional insulator, shows a distinct nonlinear feature of *I–V* curves.[[qv: 4a]],[[qv: 22c]],[[qv: 23i]] Different from C–C bonds of graphene, every C atom in fluorographene with sp^3^ hybridization is bound to an F atom. Thus, fluorographene is an insulator because of the disappearance of *π‐*conjugated bonds. Wang et al.[[qv: 23i]] investigated the typical *I–V* characteristics of fluorinated graphene prepared by CF_4_ plasma. Fluorinated graphene showed a linear *I–V* curve with a short fluorination time (<10 min) with a resistance <10 MΩ because a low amount of F and sp^3^ C atoms were regarded as defects in an sp^2^ hybridized C network. Thus, the π‐conjugated network of graphene is preserved. However, this network was destroyed by a long fluorination‐time (>10 min) using CF_4_ plasma. Fluorinated graphene underwent the transition from semiconductor to insulator, resulting in a nonlinear curve with a resistance >1 GΩ. Compared with graphene, the resistance of fluorinated graphene showed a sharp increase by more than 7 orders of magnitude (from 10 kΩ to >100 GΩ) when the fluorine coverage is a few tenths of a percent.[[qv: 23i]],^64^ Consequently, fluorinated graphene with a low F/C ratio showed a semiconducting behavior with the sp^2^ hybridized carbon network.^65^ Moreover, the insulating properties of multi‐layer fluorographene with an extremely thin thickness (5 nm) could not be changed at temperatures <400 °C, and its dielectric constant and breakdown electric field (EBD) were ≈1.2 and above 10 MV cm^−1^, respectively.^66^

### 4.5 Dispersibility

Because of the presence of C‐F bonds, fluorinated graphene and fluorographene are highly hydrophobic and difficult to disperse or solubilize in most solvents because of its low surface free energy.[[qv: 21b]],^67^ However, the dispersion of fluorographene in solvents is crucially important for the solution‐processed fabrication of devices or applications as precursors for electrodes and composites.[[qv: 26b]],[[qv: 26e]],^68^ Previous studies reported that hydrophobic fluorographene could not be dispersed in ethanol because it has no free p_z_ orbitals to form pseudohydrogen bonds with the hydroxy group of ethanol.[[qv: 13a]] The pseudohydrogen bond has been demonstrated to facilitate the dispersion of graphene with the π bond (abundant free p_z_ orbitals) in ethanol.[[qv: 13a]],[[qv: 26b]],^69^

Gong et al.[[qv: 26b]] studied the dispersibility of fluorinated graphene in a variety of organic solvents. It was found that fluorinated graphene showed much better dispersion in solvents with a large closed conjugated system formed by p_z_ orbitals such as phenylethylene (PS), NMP, and THF than others with nonhybridized p_z_ orbitals.[[qv: 26b]] Specifically, in a homogeneous solvent, a free p_z_ orbital acted as an electron acceptor and formed pseudo‐hydrogen bonds with (C*_n_*F)*_x_*–F groups, thus resulting in an increase in dispersion of fluorinated graphene.[[qv: 13a]],[[qv: 26b]] Recently, the dispersion of fluorinated graphene in water was improved by fluorosurfactants in which perfluorinated units were adhered on the surface of fluorinated graphene and cationic or anionic units provided static repulsion.^70^ Furthermore, fluorinated GO could be well dispersed in many organic solvents with nonhybridized p_z_ orbitals, such as CH_3_CN, chloroform, and DMF.[[qv: 24e]]

### 4.6 Magnetic Properties

Graphene obtained by sonochemical exfoliation of high‐purity HOPG shows a strongly diamagnetic response and no sign of ferromagnetism over a wide range of temperature, *T*.^71^ A weak sign of paramagnetism becomes noticeable only below 50 K attributed to the edge states and point defects.^71^, ^72^ Interestingly, the introduction of F atoms in graphene causes a dramatic change in magnetic properties due to the presence of C‐F bonds.^20^, ^73^ Nair et al.[[qv: 73a]] reported that the paramagnetism in the CF*_x_* samlpes with *x* increasing from 0.1 to 1 was described by the Brillouin function.

$$M=Ng\;J\mu_{B}\left[\frac{2J+1}{2J}\mathrm{ctnh}\left(\frac{2J+1}{2J}z\right)-\frac{1}{2J}\mathrm{ctnh}\left(\frac{z}{2J}\right)\right]$$

where *z = gJμ*_B_ *H/k*_B_ *T*, *g* was the *g*‐factor, *J* was the angular momentum number, *N* was the number of spins and *k*_B_ was the Boltzmann constant. The number of spins *N* increased monotonically with *x* up to ≈0.9, and then showed some decrease for fluorographene. The maximum *M* achieved by the fluorination of graphene was one order of magnitude higher than that achieved by irradiation. Unfortunately, the concentration of magnetic moments was only ≈0.1% of the maximum hypothetically possible magnetism of one moment per carbon atom because F adatoms have a strongly towards clustering.[[qv: 73a]] Tang et al.[[qv: 73b]] reported that small F clusters that could be preferably formed around the vacancies in RGO produced a lot of magnetic edge adatoms. And such fluorinated RGO have a high magnetization of 0.83 emu g^−1^, a high magnetic moment of 3.187 × 10^−3^ *μ*_B_ per carbon atom and a high efficiency of 8.68 × 10^−3^ *μ*_B_ per F adatom. Recently, fluorinated GO was used as an efficient magnetic resonance imaging (MRI) contrast agent by Ajayan et al.^20^ Tang et al.^74^ found that the uneven double‐side partially fluorinated graphene with the ripple structure become magnetic, whereas wrinkle structure showed nonmagnetic. And they also demonstrated that the magnetic moments could be significantly increased by external tensile strain.^74^

### 4.7 Tribological properties

Generally, graphene shows good tribological performance due to its high chemical inertness, extreme strength, easy shear capability on its densely packed and atomically smooth surface.^75^ Tribological properties of graphene are further improved by fluorination.^76^ Fluorinated graphene is consider as one of important ultrathin solid lubricants or lubricant additive of lubricating oils, lubricating coatings and anti‐wear composites becuase of its low friction coefficient and high durability. Specfically, fluorine atoms bound on carbon structure enhances nanoscale friction and reduces the adhesion and the number of free electrons by developing few van der Waals contacts and wide band gaps. Thus, C‐F bonding structure endows fluorinated graphene with excellent tribological performance.[[qv: 76c,d]],^77^ Carpick et al.[[qv: 76d]] systematically measured the friction between AFM tips and fluorinated graphene with different F/C ratios. This method was useful to illustrate the mechanism for the enhanced friction. They found that the enhanced friction was attributed to the significantly increased corrugation of the interfacial potential due to the highly localized negative charge concentrated at fluorine sites, consistent with the Prandtl–Tomlinson model.[[qv: 76d]] Park et al. reported that nanoscale friction on the fluorinated graphene was 6 times larger than that on pristine graphene, while the adhesion decreased somewhat becuase the attachment of F atom to the C atom enable the transition of graphene to the tetrahedral sp^3^ configuration.[[qv: 76c]],[[qv: 77a]] Hou et al.^78^ found that fluorinated graphene remarkably improved the reliability of the base oil and prolonged the friction time. Besides, trbological properties of fluorinated graphene are also controlled by its microstructures (the arrangement of F atoms, corrugation and the number of atomic layers), F/C ratios, surface chemistry (species on the surface of fluorinated graphene sheets).[[qv: 76c,d]],[[qv: 77b]],^78^ Thus, many studies need to be presented to optimize trbological properties of fluoroinated‐graphene for ultrathin solid lubricant.

### 4.8 Other properties

#### 4.8.1 Mechanical or Micromechanical Properties

Fluorination usually affects mechnical properities of graphene such as Young's modulus (*E*) and intrinsic strength (*σ*) because of the presence of C‐F bonds. Nair et al. measured the *E* and *σ* of fluorographene using AFM.[[qv: 4a]] Fluorographene exhibited a lower *E* (100 ± 30 N m^−1^) and a lower σ (≈15 N m^−1^) than of graphene (*E* and *σ* of graphene are *E* = 340 ± 50 N m^−1^ and *σ* = 42 ± 4 N m^−1^, respectively).[[qv: 4a]],^79^ They speculated that the decrease in *E* and *σ* arised from longer sp^3^ hybridized C–C bonds in fluorographene than sp^3^ hybridized C–C bonds in graphene.[[qv: 4a]] Interestingly, the elastic deformation *σ*/*E* of fluorgraphene showed litttle change in comparison of graphene because of the absence of structural defects during fluorination.[[qv: 4a]] The mechanism of controlling specifc mechanical properties including the strength, modules and deformation has yet been understood.

#### 4.8.2 Thermal Conductivity

Graphene exhibits superior thermal conductivity because of efficient phonon transfer in the 2D long‐range sp^2^ carbon framework by lattice vibrations.^80^ To date, many highly thermal conductive graphene film have been designed and prepared, and single‐ or few‐layer graphene is widely used as thermal condutive nanofiller in the polymer‐based composite to increase thermal conduction.[[qv: 80a,b,d]],^81^ Recently, fluorinated graphene shows a great promise in combing heat dissipation and hydrophobic or self‐lubricating properties. Huang et al. calculated theoretical thermal conductivity of fluorinated graphene using non‐equilibrium molecular dynamic (NEMD) simulations.^82^ Results showed that thermal conductivity of fluorinated graphene decreased during the fluorination, and it increased when the F/C ratio approached 1.0.^82^ They also found that thermal conductivity of fluorinated graphene was less sensitive to strain than of graphene. This result might be attributed to that the phonon become less sensitive to tensile strain after fluorination.^82^, ^83^ Despite great interest, improving thermal conductivity (diffusity) by exploring the key C‐F structure is one of challenge for fluorinated graphene.

## 5 Applications

### 5.1 Energy Conversion and Storage Devices

Fluorinated carbon materials (CF_x_) were first used as the cathode in lithium primary batteries by Watanabe et al. in 1972.^84^ CF_x_ was considered to be one of the ideal cathode materials for lithium primary batteries because of a variety of unique properties, such as high energy density, high average operating voltage, long shelf life, stable operation ability and wide operating temperature. Subsequently, Li/CF_x_ batteries were first commercialized by Matsushita Electric Co. in Japan in 1975.^85^ Importantly, Li/CF*_x_* batteries have the highest theoretical specific capacity (865 mA h g^−1^; *x* = 1) in primary battery systems.[[qv: 14c]],[[qv: 18c]] With an ultrathin two‐dimensional layer‐structure, fluorinated graphene and fluorographene are regarded as the most promising CF*_x_* to achieve the theoretical capacity because of their tunable F/C ratios and C‐F bonding characters, favorable diffusion kinetics of lithium ions and large specific surface area. Recently, many studies focused on the performance of Li/CF*_x_* batteries using fluorinated graphene or fluorographene as the cathode material.[[qv: 17b]],[[qv: 26c,e]],^86^

**Figure 11:**
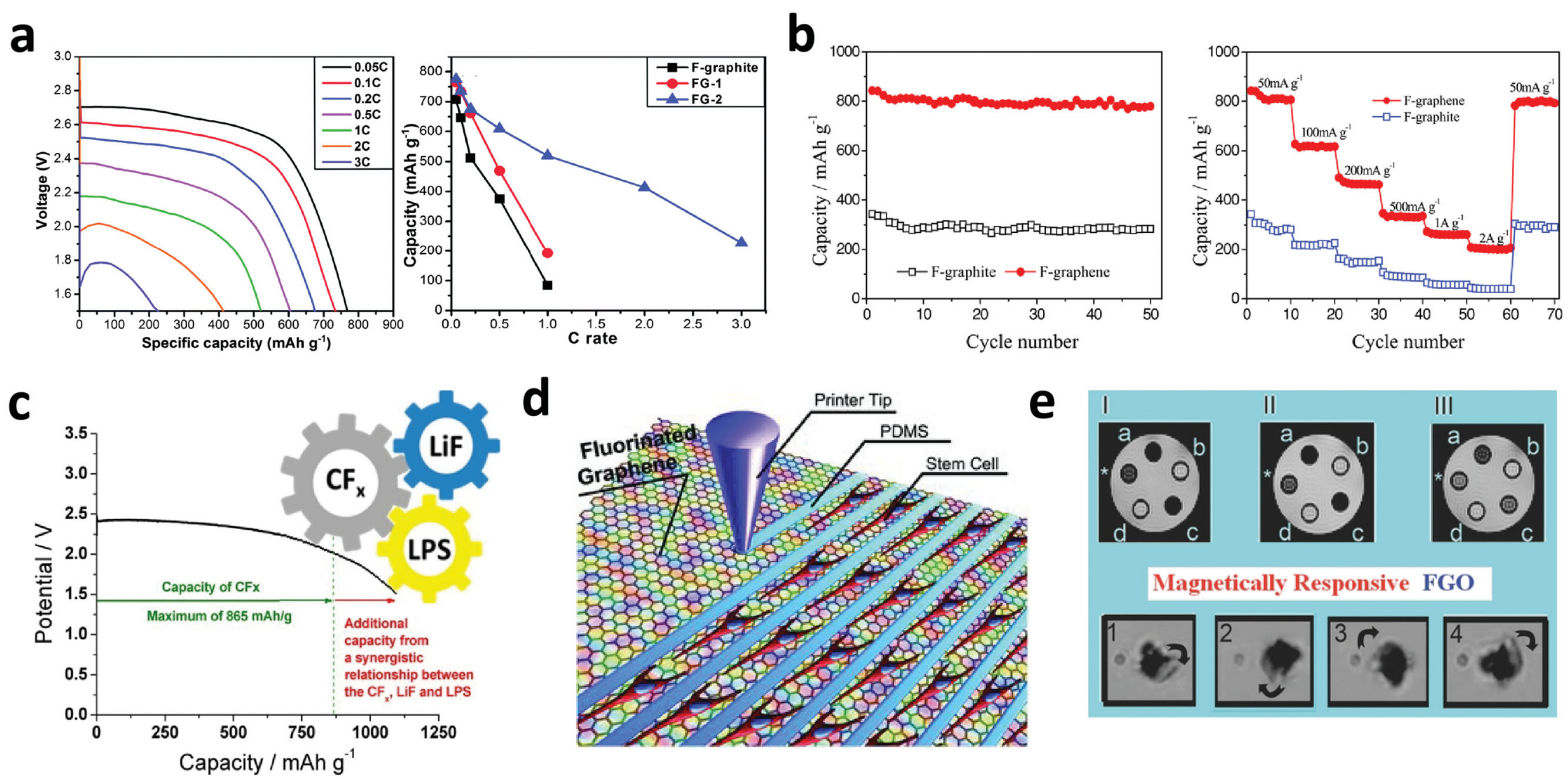
a) The galvanostatic discharge curves at different discharge rates. Reproduced with permission.[[qv: 26c]] Copyright 2014, Royal Society of Chemistry. b) Cycle performance of fluorinated graphene and fluorinated graphite electrodes at a current density of 50 mA g^−1^ and the rate and cycling performances of F‐graphene and F‐graphite electrodes obtained over a wide range of high current densities from 50 to 2000 mA g^−1^. Reproduced with permission.[[qv: 26e]] c) Discharge profile for the Li/LPS/CF*_x_* + C + LPS cell and illustrates cell capacity exceeding the theoretical maximum of 865 mAh g^−1^ for the CF*_x_* system. Reproduced with permission.[[qv: 17b]] Copyright 2014, American Chemical Society. d) Fluorinated graphene sheets as the scaffold for stem cell growth. Reproduced with permission.[[qv: 19b]] e) Magnetic properties of FGO. Reproduced with permission.^20^

Feng et al. reported that lithium primary batteries using fluorographene exhibited a remarkable discharge rate because of good Li^+^ diffusion and charge mobility through nanosheets.[[qv: 26c]] Fluorographene exfoliated by chloroform with semi‐ionic F–C bonds showed a high specific capacity of 520 mA h g^−1^ and a voltage platform of 2.18 V at a current density of 1 C, accompanied by a maximum power density of 4038 W kg^−1^ at 3 C, which was almost four times higher than that of fluorinated graphite (**Figure** **11**a).[[qv: 26c]] Moreover, fluorographene showed an energy density of 1910 Wh/kg, which is higher than fluorinated carbon nanotubes (≈1600–1800 Wh/kg).^87^ Recently, they also prepared nitrogen and fluorine co‐doped graphene with superior reversible specific discharge capacity (1075 mA h g^−1^ at 100 mA g^−1^), excellent rate capabilities (305 mA h g^−1^ at 5 A g^−1^), and outstanding cycling stability (capacity retention of ≈95% at 5 A g^−1^ after 2000 cycles) as the anode material for lithium ion batteries.^88^ Such results was attributed to the increased disorder and defects as well as the electrically conductive graphitic N and semi‐ionic C–F bonds, and the highly wrinkled nanostructures caused by the co‐doping of N and F.^88^ Zhan et al. presented a straightforward approach to fabricate self‐supporting fluorinated graphene nanosheets by liquid exfoliation of fluorinated graphite using IPA.[[qv: 26e]] Fluorinated graphene not only had abundant fluorine active sites for lithium storage but also facilitated the diffusion of lithium ions during charging and discharging. As a consequence, fluorinated graphene exhibited a high reversible capacity of 780 mAh g^−1^ at 50 mA g^−1^ and excellent cycle performance for 50 cycles (Figure 11b).[[qv: 26e]] Rangasamy et al. fabricated a solid‐state Li/CF*_x_* battery with a solid electrolyte of Li_3_PS_4_ that had dual functions: the inert electrolyte at the anode and the active CF_x_ component at the cathode.[[qv: 17b]] The solid‐state Li/CF*_x_* battery exhibited excellent capacity, good rate performance and a stable potential profile with a capacity utilization of 1095 mAh g^−1^ beyond the theoretical capacity of a CF_x_ cathode (when *x* = 1) (865 mAh g^−1^) (Figure 11c).[[qv: 17b]] In recently, Jeon et al.^89^ reported that edge‐selectively fluorinated graphene nanoplatelets (FGnPs), which prepared by mechanochemically driven reaction between fluorine gas (20 vol% in argon) and graphitic, demonstrated superb electrochemical performance with excellent stability/cycle life in lithium ion batteries. The FGnPs electrode showed an initial charge capacity of 650.3 mAh g^−1^ at 0.5 C and maintained a charge retention of 76.6% after 500 cycles.^89^ Meanwhile, the FGnPs based dye‐sensitized solar cells also displayed an outstanding performance (FF of 71.5%, *J*_sc_ of 14.44 mA cm^−2^ and PCE of 10.01%) because of the high electronegativity of F atom (*χ* = 3.98) and the strong C‐F covalent bonds (C–F, 488 kJ mol^−1^) at the edges.^89^ Results indicate that edge‐selectively fluorinated graphene is one of excellent materials for energy conversion and storage devices.

Xie et al.^90^ first reported a prototype of Mg/fluorinated graphene battery with the capacity of 110 and 90 mAh g^−1^ at 10 or 50 mA g^−1^, respectively. They utilized the fast surface redox process to replace sluggish lattice migration to improve the kinetics of Mg batteries resulting in good reversibility and rate performance. High performance benefits from the surface reaction at accessible fluorinated functional groups of porous conductive frameworks. This proof‐of‐concept Mg/fluorinated graphene system bypasses the sluggish diffusion of multivalent cations into the host lattice and the structure distortion at the cathode. Vizintin et al.^91^ used fluorinated RGO as an interlayer additive in lithium−sulfur (Li−S) batteries. Fluorinated RGO blocked the diffusion/migration of polysulfides from the porous positive electrode to the metallic lithium electrode and thus prevented the redox shuttle effect. The results showed that fluorinated RGO effectively improved the open circuit potential, cycling stability and capacitiy of Li–S batteries.

### 5.2 Bioapplications

Fluorinated graphene is of interest in many bioapplications because of its fascinating C‐F bonds that enable biological responses[[qv: 19b]],^92^ and paramagnetic behavior.^20^,[[qv: 73c]] Loh et al.[[qv: 19b]] used fluorinated graphene as the scaffold for the growth of mesenchymal stem cells (MSCs) (Figure 11d). In their study, fluorinated graphene enhanced cell adhesion and proliferation of MSCs, exhibiting a neuro‐inductive effect viaspontaneous cell polarization. Fluorinated graphene films were highly supportive of the growth of MSCs, and C‐F bonds had significant effects on cell morphology and cytoskeletal and nuclear elongation of MSCs.[[qv: 19b]] Moreover, the introduction of C‐F bonds into GO caused a dramatic change in magnetic properties. Ajayan et al.^20^ reported that fluorinated GO was an outstanding carbon‐based magnetic resonance imaging (MRI) contrast agent without magnetic nanoparticles (Figure 11e). the results showed that fluorinated GO could be potentially developed for a theranostic material with multimodal imaging, including MRI, ultrasound and photoacoustics, as well as the potential to pack hydrophobic therapeutic agents along the hydrophilic fluorinated GO basal plane.^20^,[[qv: 73c]]

### 5.3 Fluorinated Graphene Quantum Dots

Although fluorinated graphene is a semiconductor with a wide bandgap and shows UV‐fluorescence,[[qv: 12a]],[[qv: 13a]],^64^ the bundling sheets and low fluoroescent intensity restrict the application in optoelectronic devices.[[qv: 13a]],^30^, ^93^ Fluorinated graphene quantum dots (F‐GQDs) with the size <10 nm exhibits unique electronic and luminescent properties because of quantum confinement and edge effects.^94^ Tang et al. demonstrated that F‐GQDs synthesized by cutting fluorinated graphene through hydrothermal method, exhibited bright blue photoluminescence and upconversion properties.^93^ Gong et al.^95^ developed a activating‐cutting strategy to obtain graphene fluoroxide QDs with the tunable size and controllable fluorine coverage. The graphene fluoroxide QDs with good solubility and stability in water, display stable blue luminescence in hostile environment. This feature shows a great potential for the fabrication of advanced optical nano‐devices.[[qv: 95a]] Sun et al.^96^ developed a new top‐down method to simultaneously synthesize F‐GQDs and GQDs by combining a microwave‐assisted technique with the hydrothermal treatment. F‐GQDs showed excellent photo‐ and pH stability in long‐term and real‐time cellular imaging. Results open a gate to the application of fluorinated graphene in environmental engineering, solar cells, biological probes, bioimaging and energy technology. To date, the photoluminescence controlled by the defect and C‐F bonding character is still unclear.

### 5.4 Other Applications

Fluorinated graphene can also be used for applications in supercapacitors,[[qv: 24e]] electrochemistry^97^ and amphiphobicity[[qv: 21b]],^98^ applications based on unique properties controlled by F/C ratios and a two‐dimensional layer‐structure.

Zhao et al.[[qv: 24e]] prepared solid supercapacitors using fluorinated graphene as an electrode material. Cyclic voltammetry measurements showed that fluorinated graphene prepared in dichloromethane exhibited the highest specific capacitance at 106.6 F g^−1^, which was much better than GO. The results were also confirmed by charge/discharge curves.[[qv: 24e]]

Pumera et al.^97^ studied the electrochemical properties of fluorographite with three different F/C ratios of 0.33, 0.47, and 0.75. The results revealed that the heterogeneous electron transfer was accelerated by increasing F/C ratios, and the fluorographite with the F/C ratio of 0.75 showed the fastest rate of electron transfer ($k_{\mathrm{obs}}^{0}$) at 2.69 × 10^−3^ cm s^−1^ and 4.37 × 10^−3^ cm s^−1^ in [Fe(CN)_6_]^4–/3−^ and Eu^2+/3+^ redox probes, respectively. And the overpotentials of ascorbic acid and uric acid oxidations decrease with the increasing F/C ratios. And the fluorographite with F/C ratio of 0.75 provided a response to uric acid at 18.46 μA mM^−1^, which more sensitive than that to ascorbic acid (2.15 μA mM^−1^).^97^

C‐F bonds drastically reduce the surface energy of graphene, resulting in a change in wetting behavior. Mathkar et al.[[qv: 21b]] reported an amphiphobic coating of fluorinated GO with a low surface tension of 59 dyn cm^−1^, synthesized by oxidizing the basal plane of fluorinated graphite. This method allows for unique, accessible, carbon‐based amphiphobic coatings.[[qv: 21b]]

## 6 Conclusion and Outlook

In this review, we have given an overview of synthetic methods, structures and properties of fluorinated graphene that can be utilized for applications in high‐energy storage, unique biological response and magnetic resonance imaging, fluorinated graphene quantum dots, supercapacitors, electrochemistry and amphiphobicity. We have emphasized the importance and significance of controlling C‐F bonding characters, F/C ratios and configurations of fluorinated graphene, fluorographene and F‐GQDs by fluorination (gas or liquid phase) or exfoliation. The selective fluorination enables graphene with different two‐dimensional configurations for various properties including wide bandgap, blue luminescence, excellent electrochemistry, high stability and self‐lubricating. For example, an increase in the F/C ratio enlarges the bandgap of fluorographene, while a low F/C ratio usually ensures charge transport based on π‐conjugated structures. The covalent C‐F bonds in gas fluorination are crucial for thermal and chemical stability, while semi‐ionic and ionic bonds endow fluorinated graphene with a higher discharge potential for lithium batteries. Moreover, thermal conductivity, magnetic properties and luminescence of fluorinated graphene are not well developed because of a complicated fluoro‐carbon structure.

Although significant progress has been made, additional challenge for uniform up‐scale synthesis/production, target‐oriented fluorination, the homogeneity of C‐F bonding character, solution processability and removal of other fluorides in applications must be addressed. It is very difficult to tune C‐F bonding precisely at the specifc microstructure and/or chemical structure because of the strong fluorination and the complicated chemical and microstructures (layer, size, defects, configuration) of graphene. A versatile, low‐cost and safe method of fluorinating graphene has not yet been found, resulting in the limitation of a wide range of use in commercial applications. Furthermore, because the mechanism of the formation of different C‐F bonding character is still unclear, fluorinated graphene is often composed of a mixture of various types of covalent, semi‐ionic and/or ionic C‐F bonds with different ratios. As a result, fluorinated graphene with different structures (layer, size, C‐F bonds) needs to be separated and/or purified before the use in advanced electronic devices. Thus, chemical methods or strategies for selectively fluorinating graphene are of paramount importance.

To date, there is a huge number of opportunities and challenges for designing and synthesizing fluorinated graphene with different structures such as core–shell, nanoporous spheres, nanocages and topologically nontrivial assemblies. The investigation of diffenent fluorinated graphene will put insightful understanding of their properties. On the basis of controlling the selectivity of plasma‐treatment, gas‐ and/or mild fluorniation, the change in F bonding nature, bandgap or electronic interaction of fluorinated graphene with the increasing F/C ratios and/or the specific C‐F bonds will be investigated. This result will further illustrate the effect of C‐F bonding character and configuration on thermal conductivity, self‐lubricating and optical properties.

In addition to controllability and uniformity, multifunctional fluorinated graphene will be an interesting subject of intense and fruitful research in the future.A systematic study of thermal conductivity, magnetic property and luminscence will provide more special application in fluorinated graphene and F‐GQDs as well as other fluorinated carbon materials. Besides, the electrochemical properties of fluorinated graphene will be further improved to maxmize the energy density and power density by optimizing the microstructure, C‐F bonding character, the interfacial wettability and cooperation with additives. By the integration of chemical groups, polymer chains and/or functional nanoparticles, fluorinated graphene and its composites will show great potential for secondary batteries (e.g., Li, Na and Li–S battery), super‐insulating materials, light emitting diodes (LED) and display materials. Based on much effort focused on the controllability of structures and optimized properties, in the future, fluorinated graphene will exhibit excellent performance in flexible nanoelectronics, energy conversion/storage, special protective coatings, and tissue engineering.
